# Predicting Abnormal Laboratory Blood Test Results in the Intensive Care Unit Using Novel Features Based on Information Theory and Historical Conditional Probability: Observational Study

**DOI:** 10.2196/35250

**Published:** 2022-06-03

**Authors:** Camilo E Valderrama, Daniel J Niven, Henry T Stelfox, Joon Lee

**Affiliations:** 1 Data Intelligence for Health Lab Cumming School of Medicine University of Calgary Calgary, AB Canada; 2 Department of Community Health Sciences Cumming School of Medicine University of Calgary Calgary, AB Canada; 3 O’Brien Institute for Public Health Cumming School of Medicine University of Calgary Calgary, AB Canada; 4 Department of Critical Care Medicine Cumming School of Medicine University of Calgary Calgary, AB Canada; 5 Department of Cardiac Sciences Cumming School of Medicine University of Calgary Calgary, AB Canada

**Keywords:** blood laboratory test redundancy, intensive care unit, electronic medical records, machine learning, fuzzy modeling

## Abstract

**Background:**

Redundancy in laboratory blood tests is common in intensive care units (ICUs), affecting patients’ health and increasing health care expenses. Medical communities have made recommendations to order laboratory tests more judiciously. Wise selection can rely on modern data-driven approaches that have been shown to help identify low-yield laboratory blood tests in ICUs. However, although conditional entropy and conditional probability distribution have shown the potential to measure the uncertainty of yielding an abnormal test, no previous studies have adapted these techniques to include them in machine learning models for predicting abnormal laboratory test results.

**Objective:**

This study aimed to address the limitations of previous reports by adapting conditional entropy and conditional probability to extract features for predicting abnormal laboratory blood test results.

**Methods:**

We used an ICU data set collected across Alberta, Canada, which included 55,689 ICU admissions from 48,672 patients. We investigated the features of conditional entropy and conditional probability by comparing the performances of 2 machine learning approaches for predicting normal and abnormal results for 18 blood laboratory tests. Approach 1 used patients’ vitals, age, sex, and admission diagnosis as features. Approach 2 used the same features plus the new conditional entropy–based and conditional probability–based features. Both approaches used 4 different machine learning models (fuzzy model, logistic regression, random forest, and gradient boosting trees) and 10 metrics (sensitivity, specificity, accuracy, precision, negative predictive value [NPV], F_1_ score, area under the curve [AUC], precision-recall AUC, mean G, and index balanced accuracy) to assess the performance of the approaches.

**Results:**

Approach 1 achieved an average AUC of 0.86 for all 18 laboratory tests across the 4 models (sensitivity 78%, specificity 84%, precision 82%, NPV 75%, F_1_ score 79%, and mean G 81%), whereas approach 2 achieved an average AUC of 0.89 (sensitivity 84%, specificity 84%, precision 83%, NPV 81%, F_1_ score 83%, and mean G 84%). We found that the inclusion of the new features resulted in significant differences for most of the metrics in favor of approach 2. Sensitivity significantly improved for 8 and 15 laboratory tests across the different classifiers (minimum *P*<.001 and maximum *P*=.04). Mean G and index balanced accuracy, which are balanced performance metrics, also improved significantly across the classifiers for 6 to 10 and 6 to 11 laboratory tests. The most relevant feature was the pretest probability feature, which is the probability that a test result was normal when a certain number of consecutive prior tests was already normal.

**Conclusions:**

The findings suggest that conditional entropy–based features and pretest probability improve the capacity to discriminate between normal and abnormal laboratory test results. Detecting the next laboratory test result is an intermediate step toward developing guidelines for reducing overtesting in the ICU.

## Introduction

### Background

Redundancy in laboratory blood tests is common in health care [1]. Laboratory blood test redundancy increases health care expenses and reduces health care resources for future patients [1,2]. Moreover, overtesting in the intensive care unit (ICU) can harm patients’ health by causing anemia, the need for transfusion, discomfort, and poor sleep quality [3-8].

One of the areas greatly experiencing laboratory blood test redundancy is ICUs, in which daily blood tests are performed to monitor physiological functions and define clinical management strategies. Previous reports have underscored overtesting in ICUs. In a study conducted in an ICU of a tertiary hospital in Ontario, Canada, physicians retrospectively analyzed 694 blood tests performed over 4 weeks and concluded that only 48.7% of those tests were essential [9]. A similar pattern was found in a Brazilian ICU, in which approximately half (1768/3622, 48.81%) blood tests performed over 2 months resulted in normal values [10].

To reduce redundancy in the ICU, the Choosing Wisely campaign has made recommendations to order laboratory tests judiciously [11]. These recommendations have been introduced in the ICU via strategies such as education, audits and feedback, and computerized physician order entry systems [12-14]. However, these recommendations require accurate identification of laboratory tests that can be reduced without compromising the quality of patient care.

Modern data-driven approaches can help identify redundant laboratory blood tests in ICUs [15]. A study by Lee and Maslove [16] used entropy, conditional entropy, and mutual information to measure redundancy in 11 blood tests performed during the first 3 days in the ICU. They found a decreasing trend in the novelty of information throughout the ICU stay, showing that performing additional laboratory tests does not necessarily result in the gain of information. Roy et al [17] used laboratory blood test data from a tertiary academic hospital to calculate the conditional probability of a test yielding a normal result when a certain number of consecutive prior tests were already normal (pretest probability). They reported that common laboratory tests, such as those for creatinine, potassium, and sodium, had high chances of yielding normal results (>80%) when preceded by a small number (3-5) of consecutive normal results.

In addition to using data-driven approaches to describe redundancy in the ICU, other reports have used electronic medical record (EMR) data collected during the ICU stay to predict whether ordering a new blood test would provide new information. Cismondi et al [18] used heart rate, blood pressure, temperature, pulse oximeter, respiratory rate, 4 transfusion quantities, and the value of the first laboratory test performed in the day to classify redundancy among 8 different types of laboratory blood tests, with an average redundancy rate of 53%, provided to 746 patients with gastrointestinal bleeding in an ICU. To this end, they used a supervised machine learning approach with a fuzzy model, achieving an average accuracy of 79.5% for detecting redundant tests. Mahani and Pajoohan [19] followed a similar approach to predict the values of calcium and hematocrit blood tests in the same type of patients, achieving a mean absolute error of 0.03 mg/dL and 2.60%, respectively. A study by Roy et al [17] reported a maximum area under the curve (AUC) value of 0.88 for predicting low information laboratory diagnostic tests using a random forest (RF) on an extensive feature set comprising patients’ demographics, vitals, and descriptive statistics of 12 additional laboratory tests. This study was further extended by Xu et al [20], who used between 600 and 870 raw features from EMRs to predict normal laboratory results collected from 3 tertiary hospitals, achieving an area under the receiver operating characteristic curve of ≥0.90 for 12 laboratory tests.

More complex models based on deep learning have also been used to recommend laboratory reduction strategies. Yu et al [21] developed a spatial-temporal deep learning model using patients’ laboratory tests, time differences between adjacent visits, and demographics to predict the following four outputs: (1) the necessity of ordering a new laboratory test, (2) test values, (3) abnormalities (based on normal reference ranges), and (4) transitions (normal to abnormal or abnormal to normal from the latest laboratory test). By assessing different thresholds for their estimated necessity of a new test, the authors achieved a reduction rate of 20.26%, with an average abnormality or normality accuracy rate of 98.27% for 12 standard laboratory tests.

Although previous reports have shown to be effective in identifying unnecessary blood tests, none have used conditional entropy and pretest probability [16,17] to predict abnormal laboratory test results. However, as conditional entropy and pretest probability can measure the uncertainty of yielding an abnormal test for patients with different diagnoses, we hypothesize that performing feature engineering on these techniques can improve normal or abnormal laboratory test results. Feature engineering is not a common trend in data-driven approaches because of the capacity of deep learning models to learn complex and robust features from raw data. However, feature engineering is still necessary as using large amounts of raw data as input could also be a drawback as it is not always easy to obtain, clean, and process biomedical data [22]. Moreover, using additional laboratory tests as features can be counterproductive if the goal is to reduce the number of laboratory tests.

### Objectives

In this study, we adapted conditional entropy and pretest probability techniques to derive features to predict normal and abnormal laboratory test results. Our rationale is that by identifying whether the next laboratory test would yield a normal or abnormal result, medical professionals could decide on the necessity of such a test based on their experience and the patient’s diagnosis and disease severity. To evaluate the effect of the inclusion of new types of features, we compared the performance of 2 machine learning approaches for predicting normal or abnormal laboratory test results on large-scale ICU data from Alberta, Canada. The difference between the 2 approaches was that only the second approach included new features based on conditional entropy and conditional probability.

## Methods

### Alberta ICU Database

This retrospective study was conducted using the Alberta ICU data set collected from 17 ICUs, comprising 55,689 ICU admissions from 48,672 deidentified unique patients admitted between February 2012 and December 2019. The primary data source was eCritical, an EMR-based data repository containing the device and laboratory data in use in all ICUs across Alberta.

### Ethics Approval

The use of the ICU data set was approved by the Conjoint Health Research Ethics Board at the University of Calgary (reference number REB17-0389).

### Selected Laboratory Blood Tests

We focused on 18 laboratory blood tests that are common and critical in the ICU (Table 1). The reference range to determine normality was determined using the Alberta Health Services guideline [23].

**Table 1:** Blood laboratory tests and reference normal ranges for tests selected for analysis.^a^

Laboratory test			Normal range		Total records, N
Potential of hydrogen: arterial (pH)			7.20-7.40		668,388
PaO_2_^b^ (mm Hg)			70-90		668,130
PCO_2_^c^ (mm Hg)			35-45		667,889
Blood potassium (mmol/L)			3.5-5.0		400,306
**Hemoglobin (g/L)**					
	If male	140-175		398,436	
	If female	123-153		398,436	
**Blood sodium (mmol/L)**					
	If age (years) <90	136-145		396,431	
	If age (years) ≥90	132-146		396,431	
**Hematocrit (%)**					
	If male	0.42-0.50		395,046	
	If female	0.36-0.45		395,046	
White blood cells (E+9 units/L)			4.5-11.0		394,809
**Blood carbon dioxide content (mmol/L)**					
	If age (years) ≤60	23-29		390,906	
	If age (years) >60 and ≤90	23-31		390,906	
	If age (years) >90	20-29		390,906	
**Blood creatinine (µmol/L)**					
	If male and age (years) <60	80-115		370,361	
	If male and age (years) ≥60	71-115		370,361	
	If female and age (years) <60	53-97		370,361	
	If female and age (years) ≥60	53-106		370,361	
**Blood urea (µmol/L)**					
	If male and age (years) ≤55	3.0-9.0		295,445	
	If male and age (years) >55	3.0-8.0		295,445	
	If female and age (years) ≤55	3.0-8.0		295,445	
	If female and age (years) >55	2.0-7.0		295,445	
Random glucose (mmol/L)			3.3-11.0		225,627
**Alanine transaminase (U/L)**					
	If male	0-60		136,552	
	If female	0-40		136,552	
Total bilirubin (µmol/L)			1.71-20.5		133,806
Alkaline phosphatase (U/L)			40-120		128,773
Blood albumin (g/L)			30.0-45.0		102,923
**Aspartate aminotransferase (U/L)**					
	If male	10-40		98,399	
	If female	9-32		98,399	
**Gamma-glutamyl transferase (U/L)**					
	If male	0-80		36,095	
	If female	0-50		36,095	

^a^For some laboratory tests, the reference values depend on patients’ sex and age [23].

^b^PaO_2_: partial pressure of oxygen (arterial).

^c^PCO_2_: partial pressure of carbon dioxide (arterial).

### Framework Overview

This study compared 2 approaches to predict normal and abnormal blood laboratory tests performed in the ICU. The prediction was performed for all laboratory tests except for those first performed on the day, whose value was used as a feature in both approaches.

**Figure 1:**
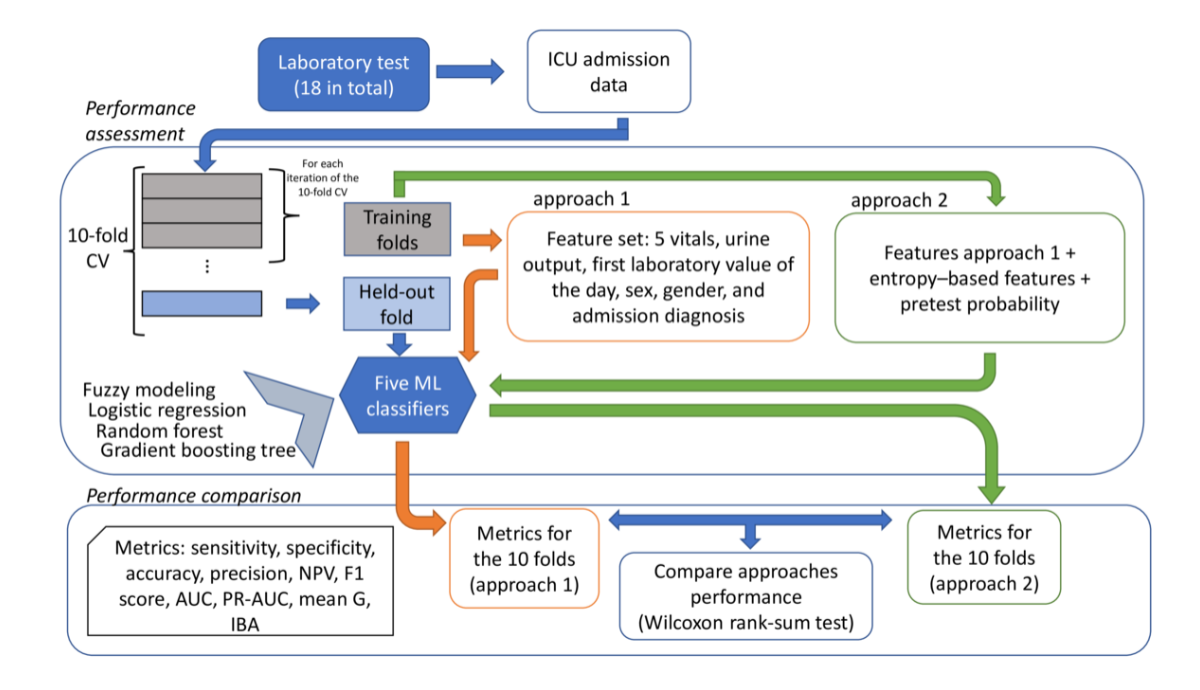
A framework to compare the two redundancy detection approaches. AUC: area under the curve; CV: cross-validation; IBA: index balanced accuracy; ICU: intensive care unit; ML: machine learning; NPV: negative predictive value; PR-AUC: precision-recall area under the curve.

Figure 1 shows an overview of the framework used to compare the 2 different approaches. Inspired by the study by Cismeondi et al [18], approach 1 used heart rate, respiration rate, heart rate, blood pressure, temperature, pulse oximeter, respiratory rate, and urine output to perform the classification. We also included additional features, namely, sex, age, and admission diagnosis. In addition to all the features from approach 1, approach 2 included the adaptation of conditional entropy and pretest probability. The following sections explain in detail the different stages of these 2 approaches. The code used for performing the comparison between the approaches is publicly available in a public repository [24]; however, our data cannot be shared because of health care regulations.

### Inclusion Criteria

ICU admissions that meet the following inclusion criteria were included in the study: aged >18 years; at least one measurement of each of heart rate, respiration rate, blood pressure, temperature, oxygen saturation, and urine output; and ≥2 orders for at least one of the 18 laboratory blood tests in Table 1.

ICU admissions satisfying these inclusion criteria were split into 10 folds. Laboratory tests from the same admission were assigned to the same fold, ensuring that ICU admissions were mutually exclusive among the folds.

### Features for Approach 1

#### Patients’ Vitals, Demographics, and Admission Diagnoses

In approach 1, the variables used to predict the abnormal results of the next laboratory blood test were heart rate (beats per minute), oxygen saturation (%), respiration rate (breaths per minute), temperature (°C), blood pressure (mm Hg), and total amount of urine void (mL). These measurements were selected as bedside monitors commonly collect large quantities of these vitals regardless of patients’ admission diagnosis. We also included patients’ sex, age, and admission diagnosis. Age and sex were included as they affect the normality of the laboratory test results (Table 1). Age corresponded to the patient’s age in years at ICU admission. The admission diagnosis was also included as patients in the ICU have a diverse set of underlying diagnoses; therefore, such a feature may affect laboratory test results. Categorical variables (sex and admission diagnosis) were coded using an approach that maps categories into numeric data using entropy, as presented in the study by Lopez-Arevalo et al [25].

#### Preprocessing Patients’ Vitals

Owing to different sampling rates, laboratory blood tests and patients’ vital measurements do not always occur simultaneously. We corrected the misalignment between laboratory tests and patients’ vitals following the steps in the study by Cismondi et al [26]. Specifically, for each admission, we fitted a cubic spline interpolation for each of the 6 patient variables (heart rate, oxygen saturation, respiration rate, temperature, blood pressure, and urine output). The patients’ vitals were then estimated at the time of the laboratory tests. The interpolation procedure used neither the laboratory test values nor their class (normal or abnormal), thus avoiding any data leakage caused by using the target predictors to preprocess the data. Moreover, as the imputation was performed per ICU admission and the 10 folds of the cross-validation procedure were mutually exclusive, imputed data were not shared between the training and test sets.

### Features for Approach 2

#### Pretest Probability

The pretest probability was calculated as the conditional probability of yielding a normal value, given a specific number of previous consecutive laboratory tests were normal. This probability was calculated on the training admissions by following the procedure presented by Roy et al [17]. Specifically, for each admission, we counted the number of consecutive normal laboratory tests before performing a new test and noted whether the new test yielded a normal result. Then, the information across the admissions was summed up, and the pretest probability distribution for each laboratory test was calculated as follows:

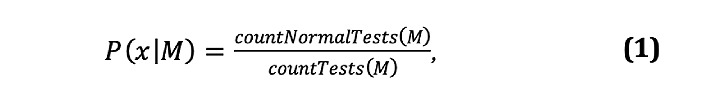



Here, *countNormalTests* is a function that returns the total cases of laboratory tests yielding normal when *M* previous tests were already normal, and *countTests* is the total number of laboratory tests performed when *M* previous consecutive tests were normal.

The pretest probability distribution was calculated using only ICU admissions from the training set. The feature values for the held-out fold were calculated using the pretest probability distribution obtained with the training folds.

#### Conditional Entropy for Abnormal Laboratory Tests

Entropy measures the expected amount of information. The conditional entropy also measures the expected amount of information of a random variable, given the occurrence of a value of secondary random variables, described as follows:

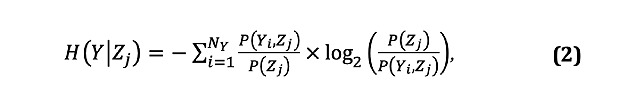



Here, *P*(*Y_i_*, *Z_j_*) is the probability of value *Y_i_* occurring while value *Z_j_* occurs, and *P*(*Z_j_*) is the probability of *Z* resulting in the possible value *Z_j_*.

We adapted conditional entropy to measure the expected amount of information of a test result if a patient’s features were already known. This conditional entropy was calculated for all the features of approach 1. The conditional entropy for each feature was calculated as follows:

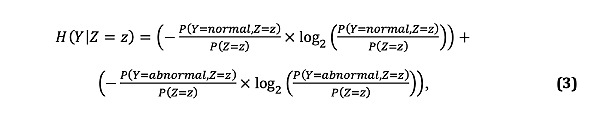



Here, *Z* is any variable of the patient’s vitals or age, *z* is a possible value for such a variable, and *Y*=normal and *Y*=abnormal indicate laboratory blood tests that yielded normal or abnormal results, respectively. The values most associated with a certain result (normal or abnormal) had lower entropy (ie, number of bits), whereas those associated with a more uncertain result had higher entropy.

To estimate the conditional probability distribution for each patient’s feature, we grouped each feature into a histogram with a bin width defined by the Freedman-Diaconis rule [27] as follows:

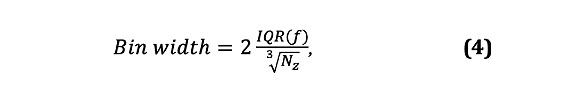



Here, IQR(*f*) is the IQR for feature *f,* and *N*_z_ is the number of observations in feature *f*.

Similar to the pretest probability, the conditional entropy distribution was calculated using only ICU admissions from the training folds. For the held-out fold, values were obtained from the distribution derived from the training folds.

### Classifiers

#### Overview

We used four different classifiers to perform the comparison between approaches 1 and 2: (1) fuzzy modeling, (2) logistic regression (LR), (3) RF, and (4) gradient boosting (GB) trees.

For all classifiers, the features of the training folds and the held-out fold set were standardized before training the models using minimum-maximum normalization to avoid any feature scale impact on the performance. Normalization was performed using the maximum and minimum values from the training set as a reference.

#### Fuzzy Model

Fuzzy models are classifiers that define rules to establish nonlinear relationships between a set of features and a response variable. In this study, we used the Takagi-Sugeno model [28], which defines rules composed of antecedents and consequences on the features as follows:

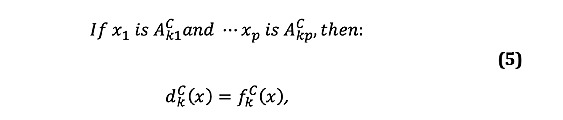



Here, *x_p_* is the *p*th feature of sample *x*; *A*_kp_^C^ is the membership function for the *k*th rule, the *p*th feature, and class *C*; and *d*_k_^C^(*x*) and *f*_k_^C^(*x*) are the discriminant and consequent for the *k*th rule and class *C*. The advantage of these rules is that they establish connectivity between the features to derive the target output. For example, a rule can state that if the heart rate is high and the first laboratory in the morning is low, the next laboratory test will be abnormal.

As multiple rules are derived for the data, they are aggregated for the final output using their degree of activation. The degree of activation of the *k*th rule for class *C* is given by the following equation:

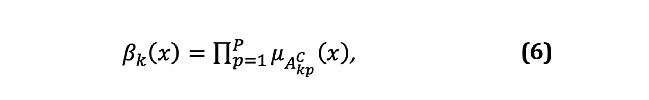



Here, 
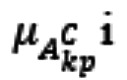
 is the membership function of the fuzzy set 
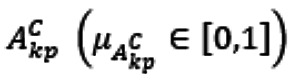
. The final discriminant for each class is as follows:

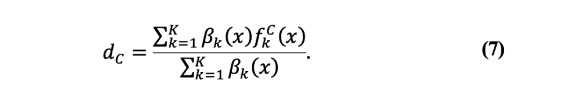



More details about fuzzy modeling can be found in the study by Takagi and Sugeno [28].

The number of features included in each rule was selected using a wrapper feature selection method that iteratively evaluated whether adding a new feature improved the model classification performance [29]. The consequence of each rule was defined using the probabilistic approach presented in the study by van den Berg et al [30] as follows:

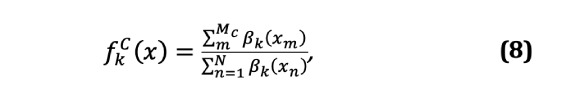



Here, 
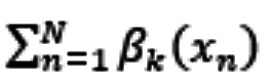
 is the summation of the degree of activation of all the samples in the training set, and 
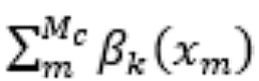
 is the summation of the degree of activation of only the samples belonging to class *C*. The fuzzy model was implemented using the Python libraries *scikit-fuzzy* [31] and *pyFume* [32].

#### Machine Learning Models

Machine learning classifiers included the LR, RF, and GB tree models. The model parameters were tuned using nested cross-validation on the grid search and defined as follows:

- For LR, the grid search for the inverse of regularization strength (*C*) was defined as {0.1, 1.0, 10.0}.
- For RF, the grid search for the number of trees was defined as {300, 500, 800}, the number of maximum splits (tree height) was defined as {8, 15, 25}, the number of minimum samples to split was defined as {5, 10}, the number of maximum samples in leaves was defined as {2, 5}, and the number of maximum features was defined as {*sqrt*, *log_2_*, None}.
- For the GB tree, the grid search for the learning rate was defined as {0.01, 0.05, 0.10}, the number of trees was defined as {300, 500, 800}, and the number of maximum features was defined as {*sqrt*, *log_2_*, None}.

The best parameters were used to retrain a model using all data from the training folds and then test the held-out fold. The models were implemented using the *sklearn* Python library [33].

### Measuring Performance

Table 2 shows the metrics used for assessing the performance of approaches 1 and 2. A total of 10 metrics were included to measure the different aspects of the approaches. Specificity, sensitivity, accuracy, and AUC measured the raw performance without considering class imbalances. In contrast, F_1_ score, AUC, precision-recall AUC, mean G, and index balanced accuracy (IBA) are less sensitive to class imbalance, thereby providing a less biased performance for assessing the approaches.

The metrics also allow the comparison of the approaches from a medical perspective. Sensitivity indicates the proportion of actual abnormal laboratory tests that were correctly classified, whereas specificity indicates the proportion of actual normal laboratory tests that were correctly classified. These 2 metrics are related to precision (positive predictive value) and negative predictive value. When the number of false positives (normal tests predicted as abnormal) increases, the specificity and precision metrics decrease. The same occurs with the sensitivity and negative predictive value metrics when the number of false negatives increases.

**Table 2:** Metrics used to measure the performance of approaches 1 and 2.

Metric	Equation	Description
Specificity	TN^a^/(FP^b^ + TN)	The proportion of actual normal laboratory tests that were correctly classified
Sensitivity (or recall)	TP^c^/(FN^d^ + TP)	The proportion of actual abnormal laboratory tests that were correctly classified
Accuracy	(TP + TN)/(FN + FP + TP + TN)	The proportion of laboratory tests that were correctly classified
Precision (positive predictive value)	TP/(FP + TP)	The proportion of laboratory tests predicted as abnormal that, in fact, were abnormal
Negative predictive value	TN/(FN + TN)	The proportion of laboratory tests predicted as normal that, in fact, were normal
F_1_ score	2 × (precision × recall)/(precision + recall)	Weighted mean of precision and recall
Area under the receiver operating characteristic curve	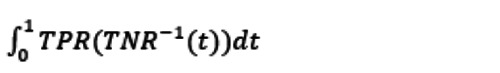	The balance between the true positive rate and true negative rate of the predictions
Area under the precision-recall curve	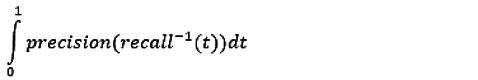	The balance between the precision and recall of the predictions
Mean G	√(sensitivity × specificity)	The balance between the performance of majority and minority classes
Index balanced accuracy [34]	(mean G)^2^ × (1 + [sensitivity – specificity])	Imbalanced index of the overall accuracy

^a^TN: true negative.

^b^FP: false positive.

^c^TP: true positive.

^d^FN: false negative.

### Comparing the 2 Approaches

The sets of metrics for each approach were compared pairwise using a 2-sided Wilcoxon rank-sum hypothesis test. The null hypothesis was that there was no difference between the metrics obtained using the 2 approaches, whereas the alternative hypothesis was that there was a difference. As 720 comparisons were conducted for the 18 laboratory tests, 4 classifiers, and 10 metrics, we used Benjamin-Hochberg correction with the false-positive rate set at 0.05.

### Relevant Features

In addition to comparing the performances of the approaches, we explored the most relevant features for classification. For each iteration of the 10-fold cross-validation, we stored the relevance of each feature for the trained model.

For each classifier, features were ranked based on their relevance values. For the fuzzy model, relevance was given by the wrapper feature selection method used to derive the antecedent of the fuzzy rules. For LR, the relevance was given by the absolute value of the coefficient associated with each feature. For the RF and GB tree, the relevance was calculated using the mean of the impurity reduction within each tree of the fitted models.

After performing the 10-fold cross-validation, a total of 10 ranking feature sets were obtained for each laboratory blood test and each classifier. We aggregated these ranking feature sets by averaging the rank of each feature, which is an aggregation strategy used in the medical domain [35,36]. Specifically, we first averaged the rank of each feature across the folds. We then aggregated the ranking features by averaging the feature rank over the classifiers. As a result, we obtained an aggregated ranking feature set for each laboratory blood test.

### Comparison Using Individual Features

To compare the performance obtained with each new feature, we compared approach 1 with the 2 alternative approaches. The first alternative approach used the features of approach 1 plus the pretest probability features, whereas the second alternative approach used the features of approach 1 plus the entropy-based features. These alternative approaches were trained and compared with the same methodology used for approaches 1 and 2.

## Results

### Performance of the Approaches

**Figure 2:**
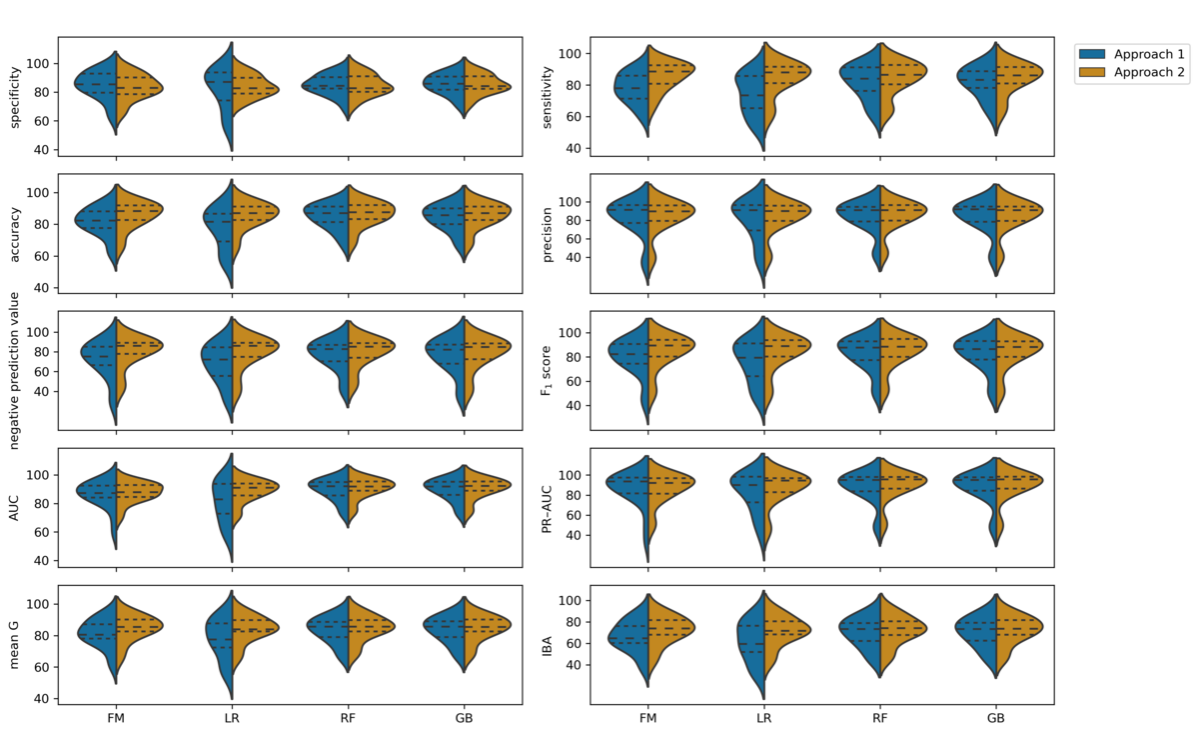
Performance distribution of approaches 1 and 2 across the laboratory blood tests. The first quantile, median, and third quantile are displayed inside each distribution (dashed lines). AUC: area under the curve; FM: fuzzy model; GB: gradient boosting; IBA: index balanced accuracy; LR: logistic regression; PR-AUC: precision-recall area under the curve; RF: random forest.

Figure 2 shows the average performance of approaches 1 and 2 across the 18 laboratory tests. Both approaches suitably predicted the laboratory blood test results. Approach 1 achieved a median performance of at most 80% for all classifiers in 5 out of the 10 metrics (specificity, accuracy, precision, AUC, and precision-recall AUC), whereas approach 2 achieved a median performance >80% for all classifiers in all metrics except IBA. Notably, higher values (>80%) in the average performance for the F_1_ score, mean G, and AUC suggest that approach 2 led to a more accurate prediction of both normal and abnormal results for most of the 18 blood laboratory tests.

The detailed performance of the approaches for each laboratory test, metric, and machine learning classifier is presented in Multimedia Appendices 1 and 2. For both approaches, the machine learning classifiers achieved similar performance. The ensemble classifiers (ie, RF and GB) achieved the best overall performance across laboratory blood tests.

### Comparison Between Old and New Features

**Figure 3:**
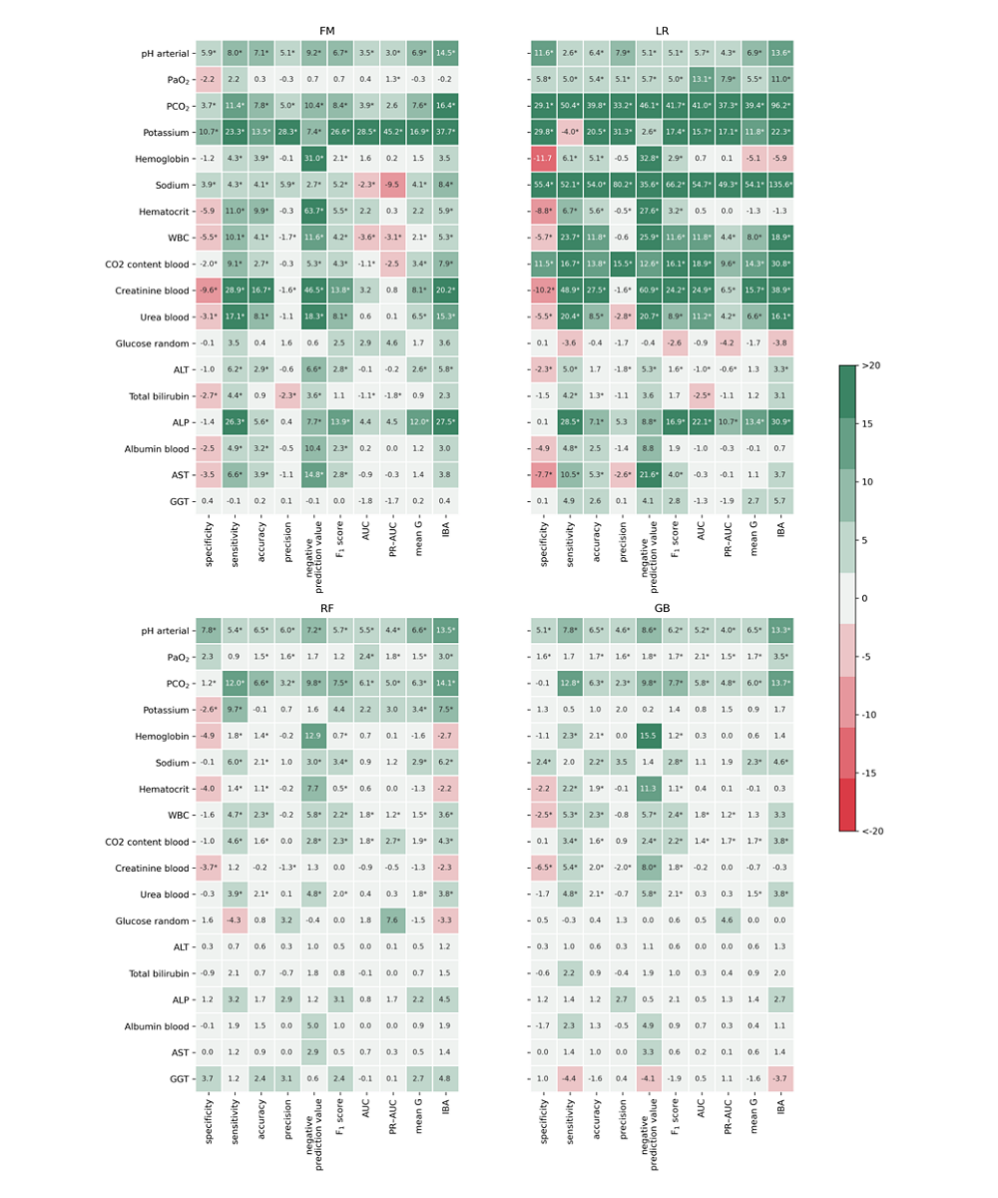
Percentage change for the 10-fold mean metric values between approaches 1 and 2. The asterisk indicates a statistically significant difference (2-sided Wilcoxon rank-sum hypothesis tests adjusted via Benjamin-Hochberg correction using a false-positive rate set at 0.05). ALP: alkaline phosphatase; ALT: alanine transaminase; AST: aspartate aminotransferase; AUC: area under the curve; FM: fuzzy model; GB: gradient boosting; GGT: gamma-glutamyl transferase; IBA: index balanced accuracy; LR: logistic regression; PaO_2_: partial pressure of oxygen (arterial); PCO_2_: partial pressure of carbon dioxide (arterial); PR-AUC: precision-recall area under the curve; RF: random forest; WBC: white blood cell.

Figure 3 shows the percentage change between approaches 1 and 2 for the 10-fold mean of each metric. The inclusion of the new features resulted in significant differences for most of the metrics in favor of approach 2. The metric that improved the most was sensitivity, achieving a significant improvement between 8 and 15 laboratory tests for the different classifiers. Specificity, in contrast, was the metric with the lowest improvement, with a significant reduction between 2 and 5 for the different classifiers. The F_1_ score, mean G, and IBA, which are balanced performance metrics, significantly improved across the classifiers, for 8 to 14, 6 to 10, and 6 to 11 laboratory tests, respectively. A detailed comparison of approaches 1 and 2 for each blood laboratory test, metric, and classifier is presented in Multimedia Appendix 3.

Among the classifiers, LR benefited the most from the inclusion of the new features, achieving an improvement of at least eight metrics for 10 out of the 18 laboratory blood tests. The RF and GB obtained less significant improvements for the different metrics than the fuzzy and LR models.

### Most Relevant Features

**Figure 4:**
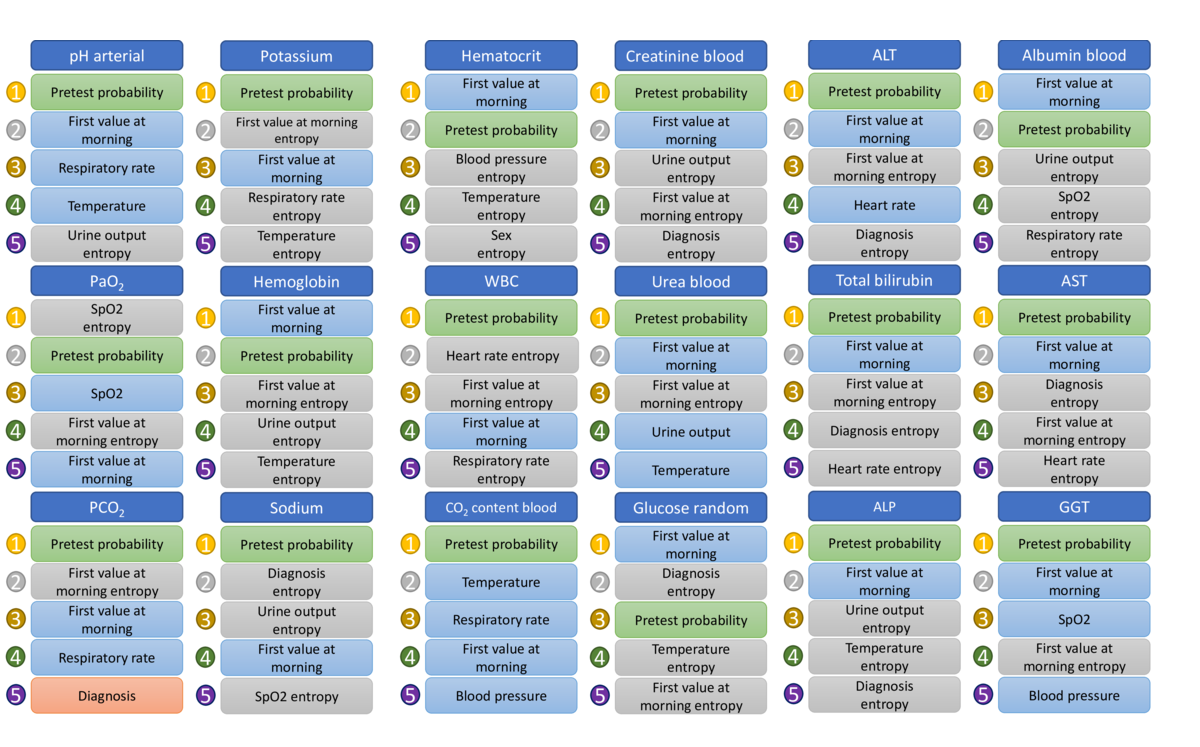
The top 5 ranking of the features selected across the machine learning classifiers for each of the laboratory tests for approach 2. Light blue- and light red boxes correspond to the vital features and diagnoses, respectively, shared with approach 1. The light green boxes correspond to the pretest probability feature, and the light gray boxes correspond to the entropy-based features. ALP: alkaline phosphatase; ALT: alanine transaminase; AST: aspartate aminotransferase; GGT: gamma-glutamyl transferase; PaO_2_: partial pressure of oxygen (arterial); PCO_2_: partial pressure of carbon dioxide (arterial); SPO2: oxygen saturation; WBC: white blood cell.

Figure 4 shows the top 5 features selected across the classifiers to discriminate between abnormal and normal laboratory blood tests for approach 2. The most common relevant feature across the laboratory tests was the pretest probability, which ranked in the first 2 positions in 17 of the 18 laboratory tests. The first value of the day was also relevant for classification, ranking in the first 2 places for half of the blood laboratory tests. The conditional entropy variant of the features, such as diagnosis, urine output entropy, respiratory entropy, and heart rate entropy, appeared more frequently in the top 5 ranking than their base forms.

Finally, to visualize how the features relate to the prediction of abnormal test results, the fuzzy predictive rules obtained by retraining a fuzzy model on the data set are presented in Multimedia Appendix 4.

### Comparison Using Individual Features

**Figure 5:**
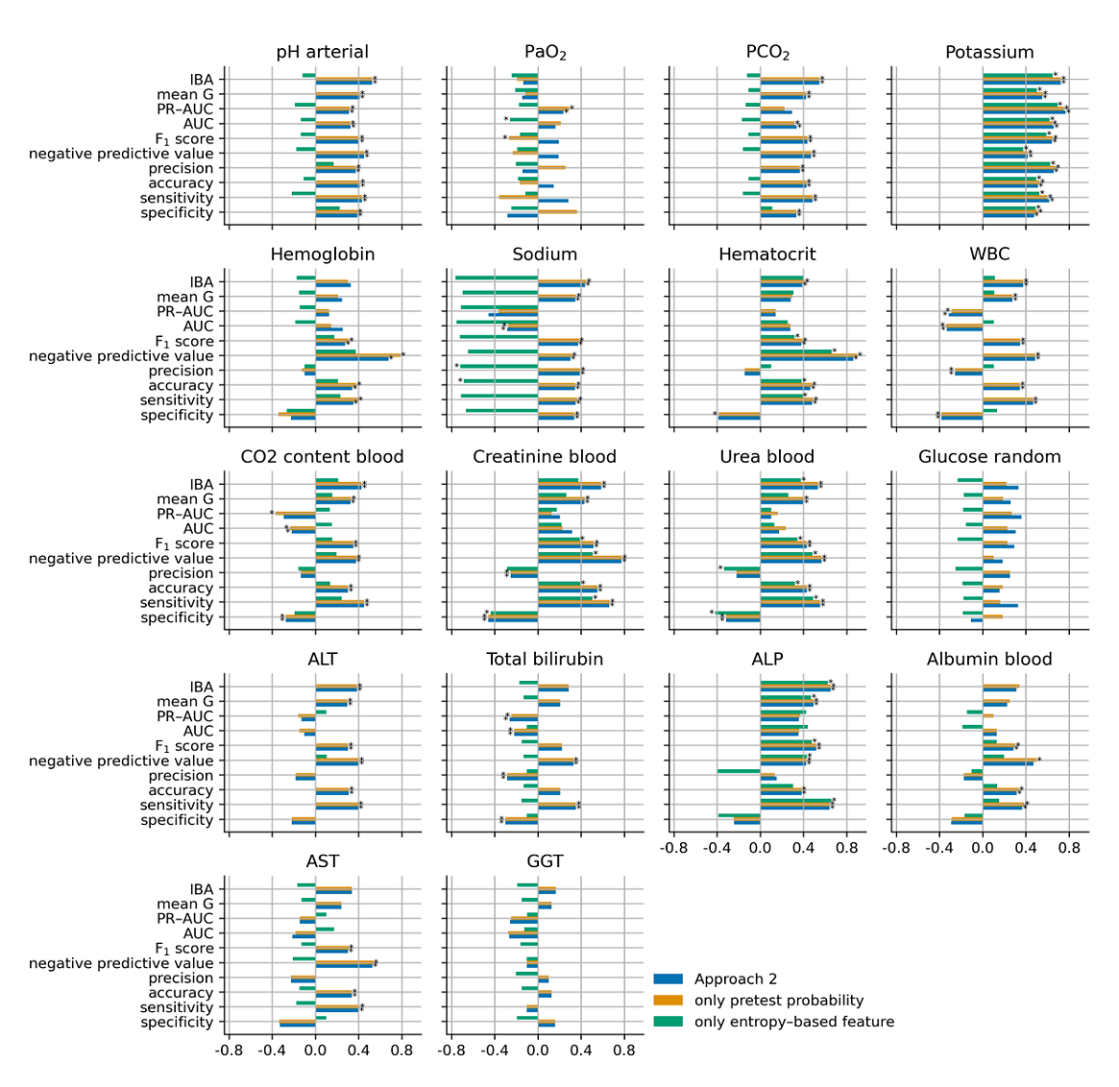
Cubic root of the percentage change between the 10-fold means of approaches 1 and 2 (blue bars), approach 1 plus the pretest probability (yellow bars), and approach 1 plus the entropy-based features for the fuzzy model. The asterisk indicates a statistically significant difference (2-sided Wilcoxon rank-sum hypothesis tests adjusted via Benjamin-Hochberg correction using a false-positive rate set at 0.05). ALP: alkaline phosphatase; ALT: alanine transaminase; AST: aspartate aminotransferase; AUC: area under the curve; GGT: gamma-glutamyl transferase; IBA: index balanced accuracy; PaO_2_: partial pressure of oxygen (arterial); PCO_2_: partial pressure of carbon dioxide (arterial); PR-AUC: precision-recall area under the curve; WBC: white blood cell.

**Figure 6:**
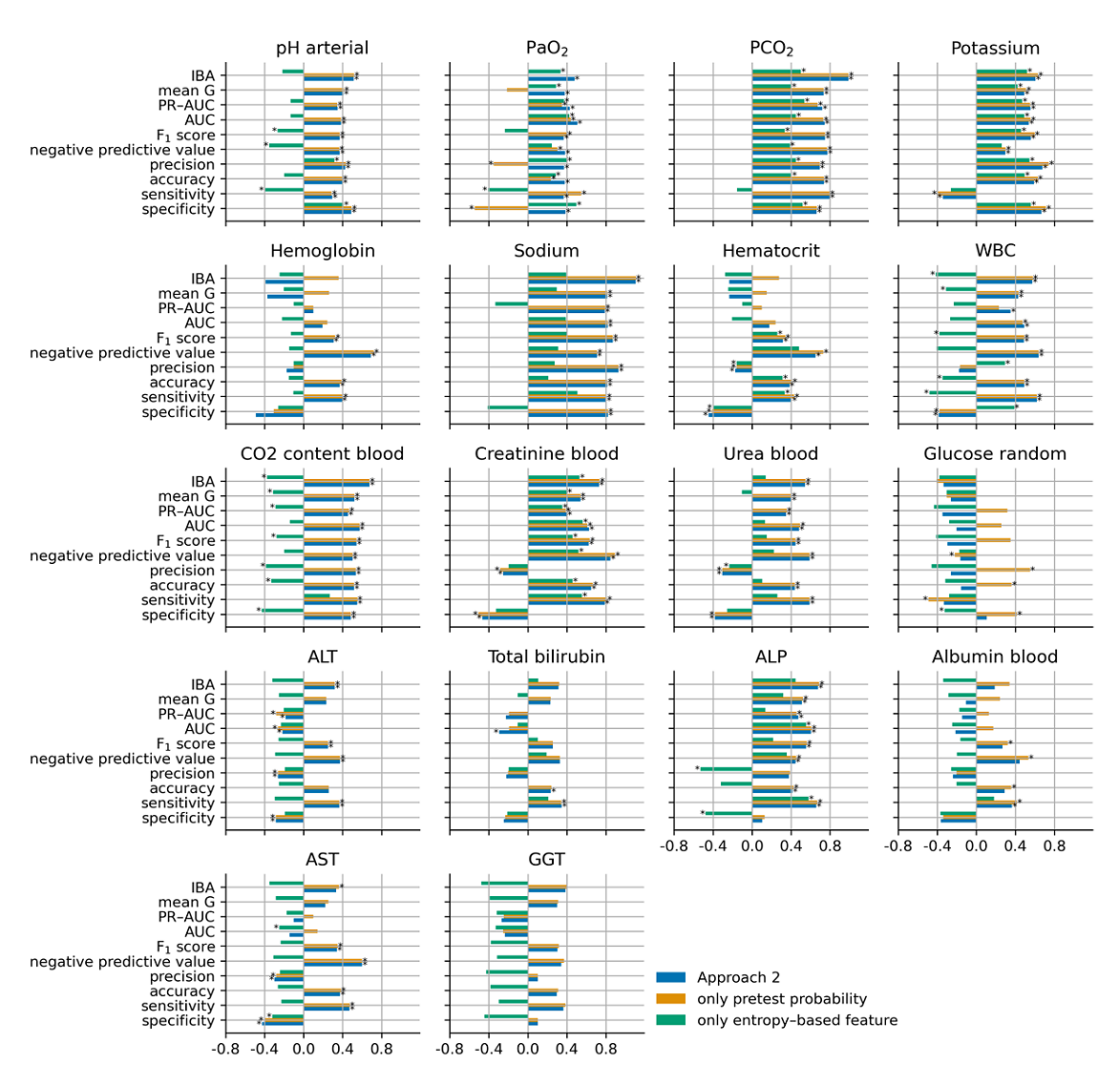
Cubic root of the percentage change between the 10-fold means of approaches 1 and 2 (blue bars), approach 1 plus the pretest probability (yellow bars), and approach 1 plus the entropy-based features for the logistic regression. The asterisk indicates a statistically significant difference (2-sided Wilcoxon rank-sum hypothesis tests adjusted via Benjamin-Hochberg correction using a false-positive rate set at 0.05). ALP: alkaline phosphatase; ALT: alanine transaminase; AST: aspartate aminotransferase; AUC: area under the curve; GGT: gamma-glutamyl transferase; IBA: index balanced accuracy; PaO_2_: partial pressure of oxygen (arterial); PCO_2_: partial pressure of carbon dioxide (arterial); PR-AUC: precision-recall area under the curve; WBC: white blood cell.

**Figure 7:**
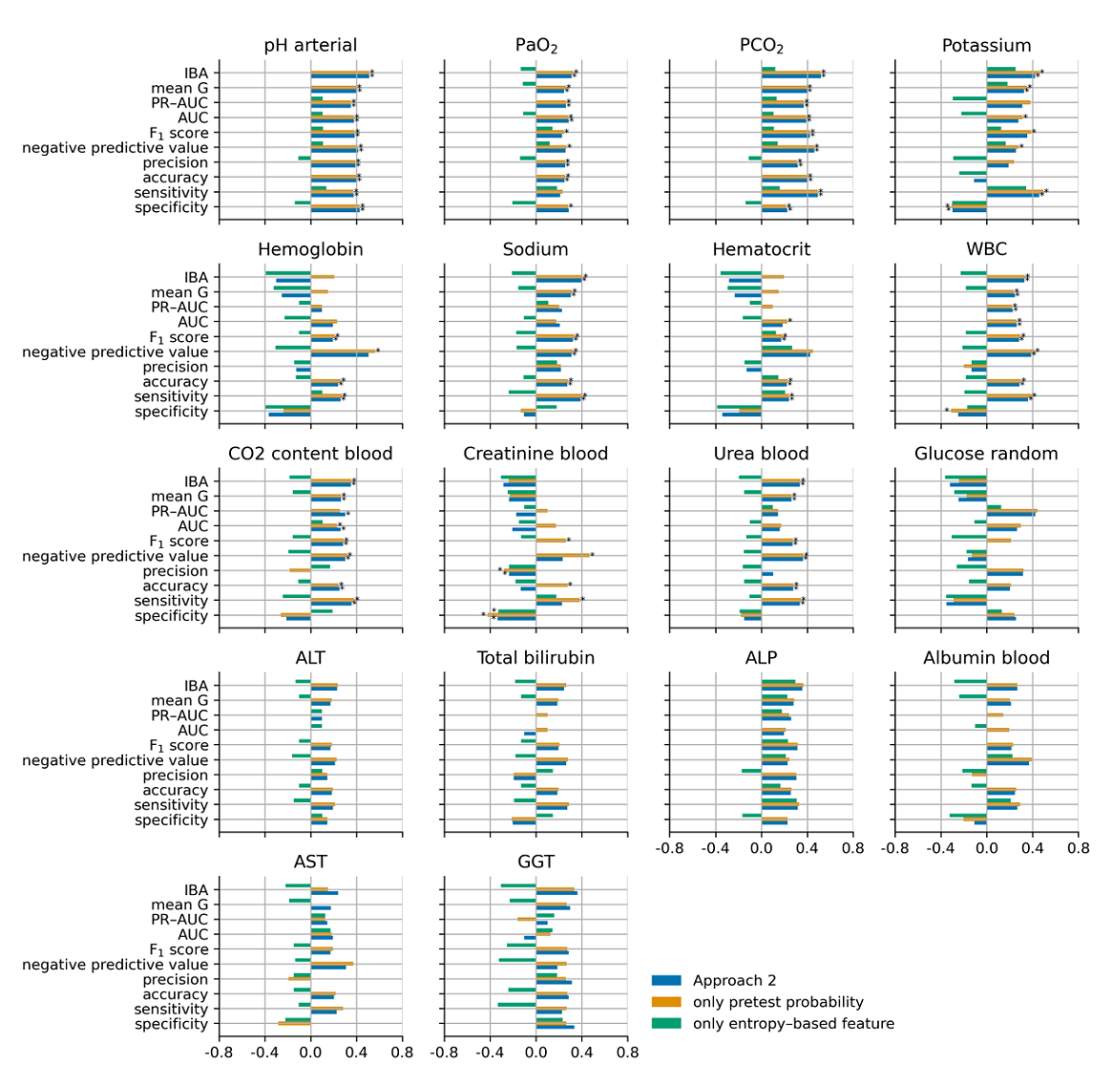
Cubic root of the percentage change between the 10-fold means of approaches 1 and 2 (blue bars), approach 1 plus the pretest probability (yellow bars), and approach 1 plus the entropy-based features for the random forest model. The asterisk indicates a statistically significant difference (2-sided Wilcoxon rank-sum hypothesis tests adjusted via Benjamin-Hochberg correction using a false-positive rate set at 0.05). ALP: alkaline phosphatase; ALT: alanine transaminase; AST: aspartate aminotransferase; AUC: area under the curve; GGT: gamma-glutamyl transferase; IBA: index balanced accuracy; PaO_2_: partial pressure of oxygen (arterial); PCO_2_: partial pressure of carbon dioxide (arterial); PR-AUC: precision-recall area under the curve; WBC: white blood cell.

**Figure 8:**
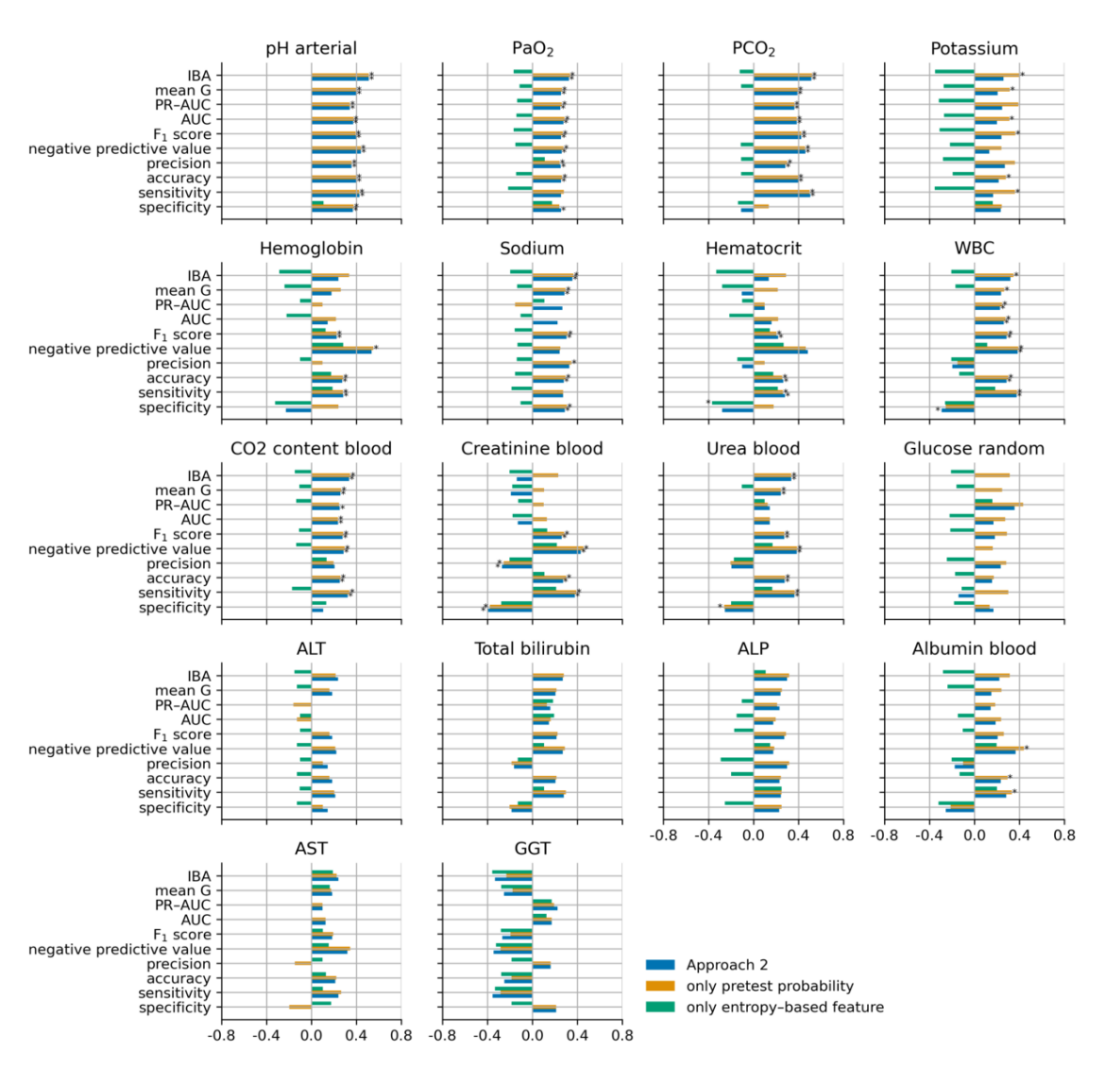
Cubic root of the percentage change between the 10-fold means of approaches 1 and 2 (blue bars), approach 1 plus the pretest probability (yellow bars), and approach 1 plus the entropy-based features for the gradient boosting model. The asterisk indicates a statistically significant difference (2-sided Wilcoxon rank-sum hypothesis tests adjusted via Benjamin-Hochberg correction using a false-positive rate set at 0.05). ALP: alkaline phosphatase; ALT: alanine transaminase; AST: aspartate aminotransferase; AUC: area under the curve; GGT: gamma-glutamyl transferase; IBA: index balanced accuracy; PaO_2_: partial pressure of oxygen (arterial); PCO_2_: partial pressure of carbon dioxide (arterial); PR-AUC: precision-recall area under the curve; WBC: white blood cell.

Figure 5, Figure 6, Figure 7, and Figure 8 show the cubic root of the percentage change between the 10-fold means of approaches 1 and 2, approach 1 plus the pretest probability feature, and approach 1 plus the entropy-based features for the fuzzy modeling, LR, RF, and GB tree, respectively. For most of the laboratory tests, the percentage change was more consistent between approach 2 and approach 1 plus the pretest probability feature. Indeed, approach 1 plus the pretest probability feature obtained the same significant improvement that was achieved with approach 2 for almost all the laboratory tests. In contrast, approach 1 plus the entropy-based features showed a negative percentage change, particularly for fuzzy and logistic models.

## Discussion

### Principal Findings

We found that the inclusion of the conditional entropy–based features and pretest probability significantly improved the capacity to predict abnormal results of a new laboratory test. Notably, the inclusion of these features improved the detection of actual abnormal tests (sensitivity) for half or more than half of the laboratory blood tests across the 4 classifiers (Figure 3).

The most relevant feature analysis revealed that the pretest probability feature was the most relevant among the new 2 types of features. In fact, the models strongly relied on the pretest probability to discriminate between normal and abnormal laboratory blood tests (Figure 4). A comparison of the performance of adding individual features further supports this fact by showing that approach 1 plus the pretest probability feature can achieve results comparable with those of approach 2.

The classifiers that improved the most were the LR and fuzzy models. A possible reason for this difference is that the LR and fuzzy models used all the features to fit their model. Instead, the ensemble models built individual trees by randomly selecting a subset of the total features, thereby excluding the pretest probabilities or entropy-based features for some trees. Nevertheless, the RF and GB tree also improved for approach 2, achieving significant improvements in the sensitivity, F_1_ score, and IBA metrics.

The inclusion of the new features improved sensitivity and negative predictive value but decreased specificity and precision. This trade-off is beneficial for the medical context because although ordering extra blood tests when it may not be necessary (higher false positives) can raise the medical expenses, patients’ safety is preserved (lower false negatives). The new features also improved balanced metrics such as F_1_ score, AUC, mean G, and IBA, thus showing the benefit of the inclusion of such types of features to improve the capacity for discriminating between normal and abnormal test results. For instance, approach 2 improved the aforementioned metrics for blood gas tests (potential of hydrogen and PCO_2_), which are among the most expensive laboratory blood tests ordered in the ICU [9].

However, we note that predicting normal and abnormal blood test results is an intermediate step toward detecting redundant tests. Deciding whether to order a new test should be based on more than predicting a normal laboratory result, as the situation and severity of each patient in the ICU are different. We included vitals and admission diagnosis to mitigate these factors; however, human interpretation still plays a crucial role in deciding whether ordering a new laboratory test is clinically meaningful. Normal laboratory test results can help measure trends, validate the required thresholds, and assess treatments. Therefore, predicting the result of a new test as normal does not imply its relevance or redundancy. However, redundancy guidelines can be established by analyzing predictions using prior consecutive results. For instance, if ≥1 previous result has yielded normal results and the prediction of the new test is again normal, the new laboratory blood test may be redundant. In contrast, if the prediction is abnormal, the new test may be relevant as it can inform medical decisions.

### Relationship With Prior Reports

The relevance of the new features is consistent with prior literature [16,17], in which entropy and conditional probability were used to describe the high redundancy that exists in ICUs. In our work, we went further by adapting these to predict the abnormal results of performing a new test. Notably, to the best of our knowledge, no previous study has used these features to predict laboratory blood test results. This study also supports the work by Cismondi et al [18], showing that patients’ vitals and the value of the first laboratory test performed in the day could guide the detection of abnormal results. However, unlike their study, we did not include their proposed blood transfusions to predict normal or abnormal laboratory test results as such transfusions targeted patients with gastrointestinal bleeding. In contrast, we extended the scope to different types of diagnoses by the inclusion of conditional entropy and pretest probability based on historical data.

In comparison with the studies by Roy et al [16] and Xu et al [20], who used machine learning to predict laboratory abnormal or normal results but did not include the pretest probability as a feature, approach 2 achieved comparable results using a smaller feature set (21 features vs 600 raw features). Specifically, the RF and GB tree achieved a mean AUC >0.89 for 13 out of the 18 laboratory tests (Multimedia Appendix 2 and Figure 2). This AUC improvement again shows the relevance of the inclusion of pretest probability as a feature in the predictive models.

### Limitations

We note that this study used an ICU data set collected in Alberta, Canada. As ethnical and racial subgroups have different distributions for laboratory tests [37], ICU data sets collected in other countries may lead to different results, particularly in low- and middle-income countries whose populations deal with economic and cultural barriers that exacerbate their health challenges. However, this study introduced new features that rely on historical data, making these features flexible and applicable to different populations. Therefore, using historical data from a different population, the conditional entropy and pretest probability distributions can be derived to calculate the uncertainty of a new test that yields an abnormal result.

We also noted that our exclusion criteria excluded patients who did not have >1 sample of the target laboratory blood test or did not have any measurements for heart rate, respiration rate, temperature, oxygen saturation, blood pressure, or urine output. This condition limits the applicability of our work as it was not designed to predict abnormal results of the first laboratory test provided in the day or when the patient’s vitals are missing. Future work should explore how to predict abnormal results of a new test in such cases.

### Conclusions

This study introduced new types of features to predict abnormal or normal results in laboratory blood tests in the ICU. The new features were extracted from historical data to describe the chances of yielding a normal test if previous sequential tests were normal (pretest probability) and the expected uncertainty of an abnormal yield if a patient’s vitals were already known (conditional entropy). These historical data combined with patients’ data are suitable indicators to predict the abnormal results of performing an additional laboratory blood test. Therefore, this study provides tools that can help develop guidelines to reduce overtesting in the ICU.

## Appendix

Multimedia Appendix 1 Performance of approach 1 using the 10-fold cross-validation for each blood laboratory test and machine learning classifier. For each classifier, the means and SDs of metrics across the 10 folds are presented. The best result for each metric and laboratory test is in bold.

Multimedia Appendix 2 Performance of approach 2 using the 10-fold cross-validation for each blood laboratory test and machine learning classifier. For each classifier, the means and SDs of metrics across the 10 folds are presented. The best result for each metric and laboratory test is in bold.

Multimedia Appendix 3 Percentage change for the 10-fold mean metric values between approach 1 and approach 2. The asterisk indicates a statistically significant difference (2-sided Wilcoxon rank-sum hypothesis tests adjusted with Benjamini-Hochberg using a false-positive rate set at 0.05). The difference was conducted for each classifier and for each metric.

Multimedia Appendix 4 Predictive rules for the fuzzy model. Entropy means that the feature is the conditional-based version.

## Abbreviations

AUC: area under the curve
EMR: electronic medical record
GB: gradient boosting
IBA: index balanced accuracy
ICU: intensive care unit
LR: logistic regression
RF: random forest
