# Supplementary material for: Predicting Abnormal Laboratory Blood Test Results in the Intensive Care Unit Using Novel Features Based on Information Theory and Historical Conditional Probability: Observational Study
Source: JMIR Med Inform. 2022 Jun 3;10(6):e35250. doi: 10.2196/35250 (PMC9206206; doi:10.2196/35250)
Supplement: Multimedia Appendix 1 [file medinform_v10i6e35250_app1.docx]

Multimedia Appendix 1. Performance of Approach 1 using 10-fold cross-validation for each blood laboratory test and machine learning classifier (FM: fuzzy model; LR: logistic regression; RF: random forest; GB: gradient boosting; BI: Bayesian inference). For each classifier, the mean (standard deviation) of metric across the ten folds is presented (Sp.: specificity; Se.: sensitivity; Ac.: accuracy; Pr.: precision; NPV: negative predictive value; F1-score; AUC; PR AUC; Gmean; IBA). The best result for each metric and laboratory test is bold.

| C | M | Sp. | Se. | Ac. | Pr. | NPV | F1 | AUC | PR AUC | G  mean | IBA |
| --- | --- | --- | --- | --- | --- | --- | --- | --- | --- | --- | --- |
| PH  Art. | FM | 78.3 (1.6) | 77.5 (0.9) | 77.9 (0.6) | 81.7 (1.6) | 73.6 (1.3) | 79.5 (0.7) | 83.9 (0.7) | 86.0 (0.9) | 77.9 (0.7) | 60.7 (1.0) |
|  | LR | 74.9 (0.9) | **80.8 (0.6)** | 78.2 (0.6) | 80.0 (1.1) | **75.8 (0.8)** | **80.4 (0.8)** | 84.3 (0.6) | 86.5 (0.7) | 77.8 (0.6) | 60.9 (0.9) |
|  | RF | 76.7 (0.9) | 79.9 (0.6) | **78.5 (0.7)** | 81.0 (1.2) | 75.4 (0.7) | **80.4 (0.8)** | 85.1 (0.6) | 87.3 (0.9) | 78.3 (0.7) | **61.5 (1.0)** |
|  | GB | **78.9 (0.9)** | 77.9 (0.6) | 78.4 (0.6) | **82.2 (1.2)** | 74.2 (0.7) | 80.0 (0.8) | **85.4 (0.6)** | **87.6 (0.4)** | **78.4 (0.7)** | **61.5 (1.0)** |
| PO2  Art. | FM | 66.8 (1.5) | 63.1 (2.4) | 64.6 (1.0) | 72.3 (0.6) | 56.8 (1.1) | 67.4 (1.5) | 69.3 (1.2) | 74.1 (0.6) | 64.9 (0.8) | 41.9 (1.2) |
|  | LR | 67.7 (1.3) | 56.0 (1.6) | 60.9 (0.7) | 70.5 (0.8) | 52.8 (0.6) | 62.4 (1.2) | 62.7 (1.0) | 72.0 (1.0) | 61.5 (0.7) | 37.4 (0.9) |
|  | RF | 68.5 (0.9) | **64.2 (0.9)** | **66.0 (0.5)** | 73.7 (0.6) | **58.1 (0.9)** | **68.6 (0.6)** | 72.0 (0.6) | 78.7 (0.5) | **66.3 (0.5)** | **43.8 (0.7)** |
|  | GB | **73.7 (1.2)** | 59.5 (1.2) | 65.5 (0.6) | **75.7 (0.7)** | 56.9 (0.7) | 66.6 (0.8) | **72.2 (0.5)** | **78.9 (3.4)** | 66.2 (0.6) | 43.2 (0.8) |
| PCO2  Art. | FM | 74.7 (2.7) | **72.1 (2.6)** | 73.3 (0.7) | 76.2 (1.6) | **70.5 (1.5)** | 74.0 (1.1) | 79.7 (0.7) | 81.4 (1.1) | 73.3 (0.6) | 53.6 (1.0) |
|  | LR | 59.8 (1.7) | 53.6 (1.3) | 56.5 (0.7) | 59.9 (1.6) | 53.4 (0.8) | 56.6 (1.1) | 59.5 (0.9) | 62.5 (1.5) | 56.6 (0.8) | 31.8 (0.8) |
|  | RF | 77.8 (0.6) | 70.9 (0.8) | 74.2 (0.4) | 78.2 (0.7) | 70.4 (0.6) | **74.4 (0.7)** | 80.9 (0.4) | 83.3 (0.8) | 74.3 (0.4) | **54.8 (0.7)** |
|  | GB | **78.8 (0.7)** | 70.2 (0.8) | **74.3 (0.4)** | **78.8 (0.7)** | 70.2 (0.6) | 74.2 (0.7) | **81.2 (0.4)** | **83.6 (9.4)** | **74.4 (0.4)** | **54.8 (0.7)** |
| K | FM | 61.6 (4.8) | 58.3 (2.3) | 60.9 (3.5) | 32.2 (2.8) | 82.6 (1.6) | 41.4 (2.5) | 58.9 (2.7) | 33.2 (2.9) | 59.9 (2.1) | 35.8 (2.4) |
|  | LR | 55.7 (2.2) | 67.0 (1.5) | 58.4 (1.5) | 31.9 (2.1) | 84.5 (1.0) | 43.2 (2.0) | 63.1 (0.7) | 36.8 (2.3) | 61.0 (1.0) | 37.7 (1.1) |
|  | RF | **76.2 (1.4)** | 59.0 (2.6) | **72.1 (1.4)** | **43.5 (2.0)** | 85.7 (1.5) | 50.0 (1.8) | **74.4 (1.3)** | **47.1 (2.5)** | 67.0 (1.6) | 44.2 (2.2) |
|  | GB | 70.0 (1.3) | **66.3 (2.4)** | 69.2 (0.8) | 40.7 (2.0) | **87.0 (1.1)** | **50.4 (1.8)** | **74.4 (1.3)** | 46.6 (3.4) | **68.1 (1.0)** | **46.2 (1.4)** |
| HGB | FM | 86.3 (4.2) | 89.0 (1.3) | 88.9 (1.3) | 99.1 (0.4) | 32.3 (3.5) | 93.8 (0.8) | 92.5 (1.7) | 99.2 (0.2) | 87.6 (2.3) | 77.0 (3.7) |
|  | LR | **87.9 (9.1)** | 86.7 (4.1) | 86.9 (3.6) | **99.2 (0.4)** | 31.4 (10.6) | 92.5 (2.1) | 93.9 (1.4) | 99.5 (0.2) | 87.1 (3.4) | 75.8 (4.9) |
|  | RF | 83.5 (3.5) | **93.0 (0.9)** | **92.5 (1.0)** | 98.9 (0.3) | **42.0 (5.1)** | **95.9 (0.6)** | **94.6 (1.4)** | **99.6 (0.1)** | 88.1 (2.1) | 78.4 (3.5) |
|  | GB | 87.1 (3.5) | 90.4 (1.1) | 90.2 (1.1) | 99.1 (0.3) | 35.5 (4.2) | 94.6 (0.6) | 94.6 (1.6) | **99.6 (0.1)** | **88.7 (2.0)** | **79.0 (3.3)** |
| Na | FM | 82.9 (2.0) | 76.7 (2.6) | 80.4 (0.9) | 74.8 (2.7) | 84.3 (0.9) | **75.7 (2.0)** | 86.6 (1.1) | 82.8 (2.6) | 79.7 (1.1) | 63.2 (1.9) |
|  | LR | 55.4 (1.9) | 52.8 (4.7) | 54.4 (2.1) | 44.0 (4.0) | 64.0 (1.4) | 48.0 (4.2) | 55.2 (4.3) | 52.3 (5.3) | 54.0 (2.6) | 29.2 (2.8) |
|  | RF | **86.2 (1.5)** | 75.6 (2.4) | **82.0 (1.2)** | **78.4 (2.7)** | 84.2 (1.0) | 77.0 (2.2) | 88.3 (1.3) | 83.7 (3.0) | 80.7 (1.5) | 64.5 (2.5) |
|  | GB | 83.6 (1.4) | **79.0 (2.1)** | 81.8 (1.2) | 76.1 (2.7) | **85.7 (1.2)** | 77.5 (2.0) | **88.4 (1.4)** | **84.1 (15.0)** | **81.2 (1.3)** | **65.7 (2.2)** |
| HCT | FM | 86.9 (4.3) | 83.6 (2.3) | 83.8 (2.0) | 98.9 (0.4) | 27.3 (3.1) | 90.6 (1.3) | 91.0 (1.9) | 99.0 (0.5) | 85.2 (1.8) | 72.4 (2.7) |
|  | LR | **90.9 (2.9)** | 85.0 (2.6) | 85.4 (2.3) | **99.2 (0.3)** | 30.1 (3.9) | 91.6 (1.5) | 93.2 (1.3) | 99.4 (0.2) | 87.8 (1.3) | 76.7 (2.2) |
|  | RF | 83.3 (3.5) | **91.8 (0.9)** | **91.3 (0.6)** | 98.7 (0.4) | **41.7 (4.2)** | **95.2 (0.4)** | **93.7 (0.7)** | **99.5 (0.1)** | 87.4 (1.6) | 77.1 (2.6) |
|  | GB | 87.5 (3.1) | 89.2 (1.0) | 89.1 (0.8) | 99.0 (0.3) | 36.3 (3.8) | 93.9 (0.5) | 93.6 (0.9) | 99.4 (0.1) | **88.4 (1.4)** | **78.2 (2.3)** |
| WBC | FM | 83.5 (2.1) | 79.3 (2.2) | 80.9 (1.2) | 89.3 (1.4) | 69.9 (1.5) | 84.0 (1.6) | 87.7 (1.1) | 92.9 (1.0) | 81.3 (1.1) | 65.9 (1.9) |
|  | LR | 83.5 (1.7) | 70.4 (2.2) | 75.2 (1.5) | 88.2 (1.0) | 61.8 (2.8) | 78.3 (1.5) | 78.7 (1.8) | 89.2 (1.1) | 76.7 (1.3) | 58.1 (2.0) |
|  | RF | 82.5 (2.0) | **81.5 (1.7)** | **81.9 (1.2)** | 89.0 (1.3) | **71.9 (2.0)** | **85.1 (1.3)** | **89.1 (1.0)** | **93.2 (0.7)** | **82.0 (1.2)** | **67.1 (1.9)** |
|  | GB | **84.7 (1.7)** | 79.5 (2.1) | 81.4 (1.4) | **90.0 (1.5)** | 70.4 (1.4) | 84.4 (1.7) | 89.0 (0.9) | **93.2 (1.8)** | **82.0 (1.3)** | 67.0 (2.3) |
| CO2 | FM | **84.0 (1.3)** | 75.7 (1.7) | 80.3 (0.7) | 79.1 (1.3) | 81.2 (1.3) | 77.4 (1.0) | 85.7 (0.8) | 82.7 (1.3) | 79.7 (0.7) | 63.0 (1.1) |
|  | LR | 73.9 (1.7) | 70.7 (2.6) | 72.5 (1.0) | 68.4 (1.2) | 75.9 (2.2) | 69.5 (1.3) | 72.6 (2.0) | 75.1 (1.3) | 72.2 (1.1) | 52.0 (1.6) |
|  | RF | 83.7 (1.4) | 78.0 (1.6) | **81.1 (0.9)** | **79.3 (1.2)** | 82.6 (1.6) | 78.6 (0.9) | 87.3 (0.8) | 83.7 (1.3) | 80.8 (0.8) | 64.8 (1.4) |
|  | GB | 82.1 (1.3) | **79.7 (1.1)** | 81.0 (0.7) | 78.1 (1.2) | **83.4 (1.3)** | **78.9 (0.8)** | **87.6 (0.9)** | **84.7 (4.8)** | **80.9 (0.7)** | **65.3 (1.1)** |
| Creat  inine | FM | 86.1 (3.1) | 71.2 (5.0) | 75.4 (3.0) | 93.1 (1.4) | 53.6 (3.0) | 80.6 (3.2) | 84.4 (2.4) | 94.2 (1.1) | 78.2 (2.3) | 60.3 (3.8) |
|  | LR | **89.3 (2.4)** | 59.9 (3.0) | 68.0 (1.9) | 93.7 (1.1) | 46.0 (1.9) | 73.0 (2.2) | 73.2 (1.7) | 90.5 (0.8) | 73.1 (1.4) | 51.9 (2.2) |
|  | RF | 85.8 (1.6) | **86.5 (1.3)** | **86.3 (1.1)** | 94.1 (0.8) | **70.8 (2.1)** | **90.1 (0.9)** | **92.8 (0.7)** | **97.2 (0.4)** | **86.1 (1.1)** | **74.3 (1.9)** |
|  | GB | 88.0 (1.2) | 83.2 (1.2) | 84.5 (1.0) | **94.8 (0.7)** | 66.6 (1.8) | 88.6 (0.8) | 92.7 (0.6) | 97.1 (3.0) | 85.5 (0.9) | 72.8 (1.6) |
| Urea | FM | 92.1 (2.0) | 78.5 (3.3) | 84.0 (1.4) | 93.6 (1.7) | 74.5 (2.9) | 85.3 (1.8) | 90.9 (2.3) | 94.8 (1.0) | 85.0 (1.4) | 71.3 (2.5) |
|  | LR | **94.4 (1.2)** | 76.3 (2.5) | 83.7 (1.1) | **95.3 (0.9)** | 73.0 (2.0) | 84.7 (1.4) | 83.8 (2.1) | 92.2 (1.1) | 84.9 (1.1) | 70.7 (2.1) |
|  | RF | 89.6 (1.5) | **88.2 (1.7)** | **88.8 (1.2)** | 92.6 (1.3) | **83.9 (1.3)** | **90.3 (1.3)** | 95.1 (0.5) | 96.7 (0.5) | 88.9 (1.2) | **79.0 (2.2)** |
|  | GB | 90.9 (1.1) | 87.3 (1.6) | 88.7 (1.0) | 93.4 (1.1) | 82.9 (1.5) | 90.2 (1.2) | **95.3 (0.5)** | **96.8 (2.1)** | **89.1 (1.0)** | **79.0 (1.9)** |
| Glucose | FM | 78.5 (3.2) | **70.8 (5.5)** | 77.4 (2.5) | 36.9 (3.4) | **93.8 (1.3)** | 48.4 (3.5) | 80.4 (2.7) | 51.7 (5.4) | 74.4 (2.5) | 55.1 (4.0) |
|  | LR | 81.9 (2.0) | 68.7 (4.0) | 80.0 (1.8) | 40.1 (3.4) | 93.7 (1.1) | 50.6 (3.6) | **82.0 (2.2)** | **52.5 (5.2)** | **74.9 (2.3)** | **55.5 (3.5)** |
|  | RF | **85.2 (1.8)** | 65.7 (4.2) | **82.4 (1.8)** | **44.0 (3.6)** | 93.4 (1.0) | **52.7 (3.8)** | 81.8 (2.0) | 49.7 (4.5) | 74.8 (2.4) | 54.9 (3.7) |
|  | GB | 81.5 (2.1) | 68.9 (4.2) | 79.7 (2.0) | 39.7 (3.8) | 93.7 (1.1) | 50.4 (4.1) | **82.0 (1.7)** | 50.1 (1.2) | 74.9 (2.5) | **55.5 (3.8)** |
| ALT | FM | 97.2 (1.2) | 89.1 (2.9) | 92.7 (1.9) | 97.5 (1.1) | 87.7 (3.3) | 93.1 (1.9) | 95.6 (1.5) | 97.4 (0.9) | 93.0 (1.8) | 85.9 (3.5) |
|  | LR | **98.5 (1.0)** | 90.1 (2.2) | 93.8 (1.6) | 98.7 (0.9) | 88.8 (3.0) | 94.2 (1.4) | 98.1 (0.5) | **98.8 (0.3)** | 94.2 (1.4) | 88.0 (2.8) |
|  | RF | 96.1 (1.8) | 93.7 (1.6) | 94.7 (1.5) | 96.7 (1.5) | 92.4 (2.4) | 95.2 (1.3) | 98.0 (0.6) | 98.6 (0.6) | 94.9 (1.5) | 89.8 (2.8) |
|  | GB | 96.2 (2.0) | **93.8 (1.5)** | **94.9 (1.4)** | **96.8 (1.7)** | **92.5 (2.0)** | **95.3 (1.3)** | **98.1 (0.5)** | 98.8 (0.1) | **95.0 (1.4)** | **90.0 (2.7)** |
| Bilirubin | FM | **95.8 (2.2)** | 85.8 (2.1) | **90.6 (1.1)** | **95.7 (1.9)** | 86.2 (2.0) | 90.5 (1.3) | 93.7 (1.0) | 95.8 (0.9) | 90.6 (1.2) | 81.4 (2.2) |
|  | LR | 94.4 (2.1) | 86.1 (2.9) | 90.1 (1.5) | 94.3 (2.0) | 86.2 (3.0) | 90.0 (1.7) | **96.3 (0.9)** | **97.1 (0.8)** | 90.2 (1.5) | 80.6 (2.9) |
|  | RF | 94.1 (2.7) | **87.5 (2.2)** | 90.7 (1.2) | 94.2 (2.3) | **87.4 (2.2)** | **90.7 (1.4)** | **96.3 (0.9)** | **97.1 (0.9)** | 90.7 (1.3) | **81.8 (2.4)** |
|  | GB | 94.0 (2.6) | 87.3 (2.9) | 90.5 (1.5) | 94.0 (2.3) | 87.2 (2.7) | 90.5 (1.8) | 95.7 (1.1) | 96.6 (0.3) | **90.5 (1.6)** | 81.4 (3.0) |
| ALP | FM | **93.2 (3.0)** | 66.5 (9.9) | 84.6 (2.1) | **83.1 (4.9)** | 85.5 (3.6) | 73.3 (5.6) | 84.8 (5.5) | 81.5 (6.2) | 78.4 (4.8) | 60.1 (8.2) |
|  | LR | 91.6 (2.1) | 64.8 (6.8) | 83.0 (2.7) | 78.7 (5.1) | 84.4 (2.8) | 71.0 (5.7) | 74.1 (5.3) | 76.0 (5.3) | 77.0 (4.2) | 57.9 (6.7) |
|  | RF | 90.6 (2.7) | 81.0 (8.2) | 87.5 (3.3) | 80.7 (4.8) | 90.8 (3.7) | 80.7 (5.6) | **92.2 (2.8)** | **87.1 (4.5)** | 85.6 (4.8) | 72.8 (8.3) |
|  | GB | 90.5 (2.6) | **83.1 (7.1)** | **88.1 (3.1)** | 80.8 (5.0) | **91.7 (3.4)** | **81.8 (5.2)** | 92.0 (3.1) | 86.9 (4.4) | **86.6 (4.2)** | **74.7 (7.4)** |
| Alb  Blood | FM | 84.9 (6.5) | 85.9 (3.0) | 85.7 (1.9) | 94.9 (2.1) | 65.1 (7.1) | 90.1 (1.5) | **92.5 (1.7)** | 97.1 (0.8) | 85.3 (2.6) | 72.9 (4.0) |
|  | LR | **86.2 (5.1)** | 84.9 (3.5) | 85.2 (2.5) | **95.3 (1.8)** | 63.9 (8.3) | 89.7 (1.8) | **92.5 (1.7)** | 97.1 (0.9) | 85.5 (2.4) | 73.0 (3.9) |
|  | RF | 82.5 (5.0) | **89.1 (2.4)** | **87.6 (1.8)** | 94.3 (1.7) | **69.9 (7.6)** | **91.6 (1.3)** | 91.8 (1.7) | **96.8 (1.1)** | **85.7 (2.4)** | **74.0 (3.9)** |
|  | GB | 84.2 (5.8) | 87.4 (2.4) | 86.7 (1.6) | 94.7 (1.9) | 67.3 (6.7) | 90.9 (1.2) | 91.6 (1.8) | 96.7 (0.4) | **85.7 (2.4)** | 73.7 (3.8) |
| AST | FM | 93.9 (2.7) | 89.4 (3.3) | 90.6 (2.2) | 97.7 (1.0) | 76.1 (5.7) | 93.3 (1.7) | 95.2 (1.7) | 98.5 (0.6) | 91.6 (1.5) | 83.5 (3.0) |
|  | LR | **97.8 (1.7)** | 85.9 (3.5) | 89.0 (2.2) | **99.1 (0.7)** | 71.4 (4.4) | 92.0 (1.9) | **97.6 (0.4)** | **99.2 (0.2)** | 91.6 (1.5) | 83.0 (3.0) |
|  | RF | 92.7 (2.7) | **93.1 (2.0)** | **93.0 (1.2)** | 97.3 (1.0) | **82.9 (3.6)** | **95.2 (0.9)** | 96.8 (1.0) | 98.9 (0.4) | **92.9 (1.1)** | **86.3 (1.9)** |
|  | GB | 92.8 (3.0) | 92.3 (2.9) | 92.5 (1.6) | 97.4 (1.0) | 81.4 (4.8) | 94.8 (1.3) | 97.4 (0.7) | 99.1 (0.2) | 92.5 (1.1) | 85.6 (2.1) |
| GGT | FM | 96.4 (5.9) | 93.1 (7.0) | 94.7 (4.9) | **96.6 (5.6)** | 93.3 (7.2) | 94.7 (5.2) | 97.8 (2.1) | 98.2 (2.2) | **94.6 (4.8)** | 89.5 (9.2) |
|  | LR | **96.7 (4.4)** | 89.4 (9.6) | 93.0 (5.0) | **96.6 (4.3)** | 90.2 (8.3) | 92.6 (5.8) | 98.8 (2.1) | **98.7 (2.5)** | 92.8 (5.3) | 85.8 (10.3) |
|  | RF | 93.9 (8.1) | 92.9 (9.1) | 93.6 (5.8) | 94.5 (6.1) | 93.7 (8.0) | 93.4 (6.5) | 98.3 (1.7) | 98.4 (2.1) | 93.2 (6.3) | 87.2 (11.8) |
|  | GB | 93.8 (8.1) | **95.7 (6.3)** | **94.8 (4.2)** | 94.5 (5.8) | **95.7 (7.0)** | **94.8 (3.9)** | **98.1 (2.0)** | 97.8 (0.0) | 94.5 (4.6) | **89.7 (8.4)** |
