# Supplementary material for: Predicting Abnormal Laboratory Blood Test Results in the Intensive Care Unit Using Novel Features Based on Information Theory and Historical Conditional Probability: Observational Study
Source: JMIR Med Inform. 2022 Jun 3;10(6):e35250. doi: 10.2196/35250 (PMC9206206; doi:10.2196/35250)
Supplement: Multimedia Appendix 2 [file medinform_v10i6e35250_app2.docx]

Multimedia Appendix 2. Performance of Approach 2 using 10-fold cross-validation for each blood laboratory test and machine learning classifier (FM: fuzzy model; LR: logistic regression; RF: random forest; GB: gradient boosting; BI: Bayesian inference). For each classifier, the mean (standard deviation) of metric across the ten folds is presented (Sp.: specificity; Se.: sensitivity; Ac.: accuracy; Pr.: precision; NPV: negative predictive value; F1-score; AUC; PR AUC; Gmean; IBA). The best result for each metric and laboratory test is in bold.

| Lab test | M | Sp. | Se. | Ac. | Pr. | NPV | F1 | AUC | PRAUC | G  mean | IBA |
| --- | --- | --- | --- | --- | --- | --- | --- | --- | --- | --- | --- |
| PH  Art. | FM | 82.9 (0.5) | 83.7 (0.5) | 83.4 (0.3) | 85.9 (0.5) | 80.4 (0.6) | 84.8 (0.5) | 86.8 (1.4) | 88.6 (1.6) | 83.3 (0.3) | 69.5 (0.6) |
|  | LR | **83.6 (0.6)** | 82.9 (0.5) | 83.2 (0.4) | **86.3 (0.6)** | 79.7 (0.6) | 84.5 (0.5) | 89.1 (0.3) | 90.2 (0.5) | 83.2 (0.4) | 69.2 (0.6) |
|  | RF | 82.7 (0.4) | **84.2 (0.5)** | **83.6 (0.4)** | 85.9 (0.6) | **80.8 (0.7)** | **85.0 (0.5)** | **89.8 (0.3)** | **91.1 (0.5)** | **83.5 (0.4)** | **69.8 (0.6)** |
|  | GB | 82.9 (0.6) | 84.0 (0.5) | 83.5 (0.4) | 86.0 (0.6) | 80.6 (0.6) | **85.0 (0.5)** | **89.8 (0.3)** | **91.1 (0.4)** | **83.5 (0.4)** | 69.7 (0.6) |
| PO2  Art. | FM | 65.3 (6.7) | 64.5 (4.0) | 64.8 (0.8) | 72.1 (2.7) | 57.2 (0.8) | 67.9 (1.2) | 69.6 (2.4) | 75.1 (3.2) | 64.7 (1.8) | 41.8 (1.9) |
|  | LR | 71.6 (1.1) | 58.8 (1.4) | 64.2 (0.5) | 74.1 (0.5) | 55.8 (0.7) | 65.5 (0.9) | 70.9 (0.5) | 77.7 (0.6) | 64.9 (0.5) | 41.5 (0.6) |
|  | RF | 70.1 (2.4) | **64.8 (2.2)** | **67.0 (0.6)** | 74.9 (1.2) | **59.1 (1.1)** | **69.4 (1.0)** | **73.7 (0.4)** | **80.1 (0.4)** | **67.3 (0.5)** | **45.1 (0.8)** |
|  | GB | **74.9 (0.8)** | 60.5 (1.0) | 66.6 (0.5) | **76.9 (0.4)** | 57.9 (0.7) | 67.7 (0.7) | **73.7 (0.4)** | **80.1 (1.1)** | **67.3 (0.4)** | 44.7 (0.6) |
| PCO2  Art. | FM | 77.5 (0.6) | **80.3 (0.5)** | 79.0 (0.3) | 80.0 (0.6) | 77.8 (0.5) | **80.2 (0.5)** | 82.8 (1.9) | 83.5 (3.7) | 78.9 (0.3) | 62.4 (0.5) |
|  | LR | 77.2 (0.6) | **80.6 (0.5)** | 79.0 (0.3) | 79.8 (0.6) | **78.0 (0.5)** | **80.2 (0.5)** | 83.9 (0.3) | 85.8 (0.6) | 78.9 (0.3) | 62.4 (0.4) |
|  | RF | **78.7 (0.5)** | 79.4 (0.4) | **79.1 (0.3)** | **80.7 (0.6)** | 77.3 (0.5) | 80.0 (0.5) | 85.8 (0.2) | 87.5 (0.6) | **79.0 (0.3)** | **62.5 (0.4)** |
|  | GB | **78.7 (0.8)** | 79.2 (0.6) | 79.0 (0.3) | 80.6 (0.6) | 77.1 (0.5) | 79.9 (0.5) | **85.9 (0.2)** | **87.6 (0.7)** | 78.9 (0.3) | 62.3 (0.5) |
| K | FM | 68.2 (1.3) | **71.9 (1.7)** | 69.1 (0.9) | 41.3 (2.2) | **88.7 (0.9)** | **52.4 (2.0)** | 75.7 (1.4) | 48.2 (2.7) | **70.0 (0.9)** | **49.3 (1.3)** |
|  | LR | 72.3 (1.4) | 64.3 (2.5) | 70.4 (1.0) | 41.9 (1.4) | 86.7 (1.4) | 50.7 (1.4) | 73.0 (1.3) | 43.1 (1.8) | 68.2 (1.2) | 46.1 (1.7) |
|  | RF | **74.2 (1.5)** | 64.7 (1.8) | **72.0 (1.1)** | **43.8 (1.7)** | 87.1 (1.2) | 52.2 (1.3) | **76.0 (1.2)** | **48.5 (1.9)** | 69.3 (0.9) | 47.5 (1.3) |
|  | GB | 70.9 (1.5) | 66.6 (2.7) | 69.9 (1.1) | 41.5 (1.5) | 87.2 (1.4) | 51.1 (1.5) | 75.0 (1.2) | 47.3 (1.9) | 68.7 (1.3) | 47.0 (1.8) |
| HGB | FM | 85.3 (4.2) | 92.8 (1.9) | 92.4 (1.8) | 99.0 (0.3) | 42.3 (4.8) | 95.8 (1.1) | 94.0 (2.1) | 99.4 (0.3) | 88.9 (2.3) | 79.7 (3.8) |
|  | LR | 77.6 (21.9) | 92.0 (3.1) | 91.3 (2.1) | 98.7 (0.9) | 41.7 (12.8) | 95.2 (1.3) | 94.6 (1.2) | 99.6 (0.2) | 82.7 (15.5) | 71.3 (19.2) |
|  | RF | 79.4 (4.1) | **94.7 (0.6)** | **93.8 (0.7)** | 98.7 (0.3) | **47.4 (4.9)** | **96.6 (0.4)** | **95.3 (1.3)** | **99.7 (0.1)** | 86.7 (2.3) | 76.3 (3.8) |
|  | GB | **86.1 (3.2)** | 92.5 (1.0) | 92.1 (1.0) | **99.1 (0.3)** | 41.0 (4.6) | 95.7 (0.6) | 94.9 (1.7) | 99.6 (0.1) | **89.2 (1.6)** | **80.1 (2.7)** |
| Na | FM | **86.1 (1.0)** | 80.0 (1.1) | 83.7 (0.7) | 79.2 (1.9) | 86.6 (0.8) | 79.6 (1.3) | 84.6 (1.1) | 74.9 (11.3) | 83.0 (0.8) | 68.5 (1.3) |
|  | LR | **86.1 (0.9)** | 80.3 (1.5) | **83.8 (0.8)** | **79.3 (1.9)** | 86.8 (0.7) | **79.8 (1.6)** | 85.4 (1.1) | 78.1 (3.8) | **83.2 (0.9)** | **68.8 (1.6)** |
|  | RF | **86.1 (1.1)** | 80.1 (1.6) | 83.7 (0.8) | 79.2 (2.0) | 86.7 (0.8) | 79.6 (1.6) | 89.1 (1.2) | 84.7 (3.0) | 83.0 (1.0) | 68.5 (1.7) |
|  | GB | 85.6 (1.1) | **80.6 (1.4)** | 83.6 (1.0) | 78.8 (2.1) | **86.9 (0.7)** | 79.7 (1.7) | **89.4 (1.2)** | **85.7 (2.2)** | 83.1 (1.0) | 68.7 (1.7) |
| HCT | FM | 81.8 (4.8) | 92.8 (1.6) | 92.1 (1.3) | 98.6 (0.5) | 44.7 (6.3) | 95.6 (0.8) | 93.0 (2.0) | 99.3 (0.4) | 87.1 (2.3) | 76.7 (3.7) |
|  | LR | 82.9 (5.0) | 90.7 (0.7) | 90.2 (0.5) | 98.7 (0.5) | 38.4 (3.5) | 94.5 (0.3) | 93.7 (1.3) | 99.4 (0.2) | 86.7 (2.4) | 75.7 (3.8) |
|  | RF | 80.0 (4.1) | **93.1 (0.7)** | **92.3 (0.6)** | 98.5 (0.4) | **44.9 (4.0)** | **95.7 (0.4)** | **94.3 (1.4)** | **99.5 (0.2)** | 86.3 (2.2) | 75.4 (3.5) |
|  | GB | **85.6 (3.9)** | 91.2 (0.8) | 90.8 (0.8) | **98.9 (0.4)** | 40.4 (4.2) | 94.9 (0.4) | 94.0 (1.3) | **99.5 (0.0)** | **88.3 (2.1)** | **78.4 (3.4)** |
| WBC | FM | 78.9 (1.4) | **87.3 (0.9)** | **84.2 (0.6)** | 87.8 (0.9) | **78.0 (1.7)** | **87.5 (0.7)** | 84.5 (0.9) | 90.0 (2.5) | 83.0 (0.7) | 69.4 (1.0) |
|  | LR | 78.7 (1.4) | 87.1 (0.9) | 84.1 (0.6) | 87.7 (0.8) | 77.8 (1.8) | 87.4 (0.7) | 88.0 (0.9) | 93.1 (0.8) | 82.8 (0.6) | 69.1 (1.0) |
|  | RF | 81.2 (1.2) | 85.3 (1.8) | 83.8 (1.0) | 88.8 (0.8) | 76.1 (2.0) | 87.0 (1.2) | **90.7 (0.7)** | **94.3 (0.6)** | **83.2 (0.7)** | **69.5 (1.3)** |
|  | GB | **82.6 (1.4)** | 83.7 (1.1) | 83.3 (0.7) | **89.3 (0.9)** | 74.4 (1.8) | 86.4 (0.8) | 90.6 (0.7) | **94.3 (0.5)** | 83.1 (0.7) | 69.2 (1.1) |
| CO2 | FM | 82.3 (1.3) | **82.6 (1.4)** | **82.5 (0.9)** | 78.9 (1.2) | **85.5 (1.2)** | **80.7 (1.1)** | 84.8 (0.8) | 80.6 (3.8) | 82.4 (0.9) | **68.0 (1.6)** |
|  | LR | 82.4 (1.3) | 82.5 (1.4) | **82.5 (0.9)** | 79.0 (1.2) | **85.5 (1.2)** | **80.7 (1.2)** | 86.3 (0.6) | 82.3 (1.2) | **82.5 (1.0)** | **68.0 (1.6)** |
|  | RF | **82.9 (1.3)** | 81.6 (1.7) | 82.4 (0.8) | **79.3 (1.4)** | 84.9 (1.4) | 80.4 (1.1) | **88.9 (0.6)** | 86.0 (0.9) | 82.3 (0.9) | 67.6 (1.5) |
|  | GB | 82.2 (1.4) | 82.4 (1.4) | 82.3 (0.8) | 78.8 (1.3) | 85.4 (1.0) | 80.6 (1.1) | 88.8 (0.7) | **86.1 (1.4)** | 82.3 (0.8) | 67.8 (1.3) |
| Creat  inine | FM | 77.8 (2.6) | **91.8 (1.3)** | **88.0 (1.1)** | 91.6 (0.9) | **78.5 (2.6)** | 91.7 (0.8) | 87.1 (2.6) | 95.0 (1.4) | 84.5 (1.4) | 72.5 (2.2) |
|  | LR | 80.2 (2.4) | 89.2 (1.3) | 86.7 (0.7) | 92.2 (0.8) | 74.0 (2.2) | **90.7 (0.6)** | 91.4 (0.6) | 96.4 (0.4) | 84.6 (1.0) | 72.1 (1.5) |
|  | RF | **82.6 (1.5)** | 87.5 (1.9) | 86.1 (1.3) | 92.9 (0.5) | 71.7 (3.2) | 90.1 (1.0) | 92.0 (1.0) | 96.7 (0.5) | **85.0 (0.9)** | **72.6 (1.6)** |
|  | GB | 82.3 (2.2) | 87.7 (1.2) | 86.2 (0.8) | **92.9 (0.8)** | 71.9 (1.6) | 90.2 (0.7) | **92.5 (0.6)** | **97.1 (0.2)** | 84.9 (1.0) | **72.6 (1.5)** |
| Urea | FM | 89.2 (1.2) | **91.9 (0.7)** | 90.8 (0.6) | 92.6 (1.0) | **88.1 (0.9)** | **92.2 (0.7)** | 91.4 (1.4) | 94.9 (0.9) | **90.5 (0.6)** | **82.2 (1.1)** |
|  | LR | 89.2 (1.2) | **91.9 (0.7)** | **90.8 (0.6)** | 92.6 (1.0) | **88.1 (0.9)** | **92.2 (0.7)** | 93.2 (0.6) | 96.1 (0.4) | **90.5 (0.6)** | 82.1 (1.1) |
|  | RF | 89.3 (1.3) | 91.6 (1.1) | 90.7 (0.6) | **92.7 (1.0)** | 87.9 (1.0) | 92.1 (0.7) | 95.5 (0.6) | 97.0 (0.6) | **90.5 (0.6)** | 82.0 (1.0) |
|  | GB | **89.4 (1.2)** | 91.5 (0.6) | 90.6 (0.6) | **92.7 (1.1)** | 87.7 (0.9) | 92.1 (0.7) | **95.6 (0.3)** | **97.1 (0.7)** | 90.4 (0.7) | 82.0 (1.2) |
| Glucose | FM | 78.4 (2.6) | **73.3 (4.6)** | 77.7 (1.8) | 37.5 (3.2) | **94.4 (0.8)** | 49.6 (3.6) | 82.7 (1.6) | **54.1 (5.1)** | **75.7 (1.9)** | **57.1 (3.1)** |
|  | LR | 82.0 (1.9) | 66.2 (4.3) | 79.7 (1.4) | 39.4 (3.6) | 93.3 (0.7) | 49.3 (3.8) | 81.3 (1.9) | 50.3 (5.7) | 73.6 (1.9) | 53.4 (3.0) |
|  | RF | **86.6 (2.0)** | 62.9 (5.1) | **83.1 (1.6)** | **45.4 (3.3)** | 93.0 (0.7) | **52.7 (3.9)** | **83.3 (1.7)** | 53.5 (4.5) | 73.7 (2.6) | 53.1 (4.0) |
|  | GB | 81.9 (2.4) | 68.7 (4.0) | 80.0 (1.7) | 40.2 (2.9) | 93.7 (0.6) | 50.7 (3.4) | 82.4 (1.6) | 52.4 (2.5) | 74.9 (1.7) | 55.5 (2.7) |
| ALT | FM | 96.2 (1.6) | 94.6 (1.3) | 95.4 (1.1) | 96.9 (1.4) | **93.5 (1.5)** | 95.7 (1.1) | 95.5 (1.2) | 97.2 (0.9) | 95.4 (1.1) | 90.9 (2.1) |
|  | LR | 96.2 (1.6) | 94.6 (1.3) | 95.4 (1.1) | 96.9 (1.4) | **93.5 (1.5)** | 95.7 (1.1) | 97.1 (0.8) | 98.2 (0.6) | 95.4 (1.1) | 90.9 (2.1) |
|  | RF | 96.4 (1.7) | 94.4 (1.3) | 95.3 (1.1) | 97.0 (1.3) | 93.3 (1.7) | 95.7 (1.1) | 98.0 (0.5) | 98.7 (0.4) | 95.4 (1.2) | 90.9 (2.2) |
|  | GB | **96.5 (1.7)** | **94.7 (1.3)** | **95.5 (1.2)** | **97.1 (1.3)** | **93.5 (1.7)** | **95.9 (1.1)** | **98.1 (0.5)** | **98.8 (0.3)** | **95.6 (1.2)** | **91.2 (2.3)** |
| Bilirubin | FM | 93.2 (1.5) | 89.6 (2.0) | **91.4 (1.0)** | 93.5 (1.5) | **89.3 (1.7)** | **91.5 (1.2)** | 92.7 (0.7) | 94.1 (1.5) | **91.4 (1.0)** | **83.3 (1.9)** |
|  | LR | 93.0 (1.4) | **89.7 (2.0)** | 91.3 (0.8) | 93.3 (1.2) | **89.3 (1.7)** | **91.5 (1.1)** | 93.9 (2.1) | 96.0 (1.2) | 91.3 (0.8) | 83.1 (1.6) |
|  | RF | 93.3 (1.9) | 89.3 (2.3) | 91.3 (1.1) | 93.5 (1.6) | 89.0 (1.8) | 91.4 (1.4) | **96.2 (1.0)** | **97.1 (0.9)** | 91.3 (1.2) | 83.0 (2.3) |
|  | GB | **93.4 (1.5)** | 89.2 (2.1) | 91.3 (1.0) | **93.6 (1.5)** | 88.9 (1.9) | 91.4 (1.3) | 96.0 (1.0) | 97.0 (0.5) | 91.3 (1.1) | 83.0 (2.1) |
| ALP | FM | **91.9 (2.1)** | 84.0 (6.3) | **89.3 (2.7)** | **83.4 (4.0)** | 92.1 (3.2) | **83.5 (4.3)** | 88.5 (3.5) | 85.2 (3.9) | **87.8 (3.7)** | 76.6 (6.7) |
|  | LR | 91.7 (2.4) | 83.3 (6.0) | 88.9 (2.7) | 82.9 (4.0) | 91.8 (3.1) | 83.0 (4.1) | 90.5 (3.0) | 84.1 (5.2) | 87.3 (3.6) | 75.8 (6.4) |
|  | RF | 91.7 (2.2) | 83.6 (6.6) | 89.0 (2.8) | 83.0 (4.0) | 91.9 (3.4) | 83.2 (4.3) | **92.9 (3.0)** | **88.6 (4.2)** | 87.5 (3.8) | 76.1 (6.9) |
|  | GB | 91.6 (2.7) | **84.3 (6.1)** | 89.2 (2.8) | 83.0 (4.2) | **92.2 (3.2)** | **83.5 (4.1)** | 92.5 (2.6) | 88.0 (4.8) | **87.8 (3.6)** | **76.7 (6.4)** |
| Alb  Blood | FM | **82.8 (5.9)** | **90.1 (2.7)** | 88.4 (2.4) | **94.4 (2.1)** | 71.9 (8.4) | 92.2 (1.7) | **92.7 (1.6)** | **97.1 (0.8)** | 86.3 (3.3) | 75.1 (5.4) |
|  | LR | 82.0 (5.1) | 89.0 (2.6) | 87.3 (2.5) | 94.0 (2.1) | 69.5 (7.4) | 91.4 (1.8) | 91.6 (2.1) | 96.8 (1.1) | 85.4 (3.1) | 73.5 (5.1) |
|  | RF | 82.4 (5.5) | 90.8 (2.0) | **88.9 (2.1)** | 94.3 (2.0) | **73.4 (6.0)** | **92.5 (1.6)** | 91.8 (1.8) | 96.8 (1.2) | **86.5 (3.1)** | 75.4 (5.0) |
|  | GB | **82.8 (5.8)** | 89.4 (2.1) | 87.8 (2.3) | 94.2 (2.3) | 70.6 (6.0) | 91.7 (1.8) | 92.2 (2.0) | 97.0 (0.5) | 86.0 (3.3) | **74.5 (5.3)** |
| AST | FM | 90.6 (3.1) | **95.3 (2.0)** | **94.1 (1.3)** | 96.6 (1.1) | **87.4 (4.4)** | **95.9 (1.0)** | 94.3 (1.7) | 98.2 (0.6) | 92.9 (1.4) | 86.7 (2.5) |
|  | LR | 90.3 (3.8) | 94.9 (2.5) | 93.7 (1.5) | 96.5 (1.5) | 86.8 (4.2) | 95.7 (1.2) | 97.3 (0.6) | 99.1 (0.3) | 92.6 (1.6) | 86.1 (2.8) |
|  | RF | 92.7 (2.2) | 94.2 (2.2) | 93.8 (1.3) | 97.3 (0.9) | 85.3 (4.7) | 95.7 (1.0) | 97.5 (0.6) | **99.2 (0.3)** | **93.4 (0.9)** | **87.5 (1.7)** |
|  | GB | **92.8 (3.5)** | 93.6 (2.6) | 93.4 (1.5) | **97.4 (1.2)** | 84.1 (4.9) | 95.4 (1.1) | **97.6 (0.6)** | **99.2 (0.1)** | 93.1 (1.3) | 86.8 (2.3) |
| GGT | FM | **96.8 (5.2)** | 93.0 (7.1) | 94.9 (5.3) | **96.7 (5.7)** | 93.2 (7.4) | 94.7 (5.7) | 96.0 (5.4) | 96.5 (5.0) | 94.8 (5.3) | 89.9 (10.0) |
|  | LR | **96.8 (5.2)** | 93.8 (5.3) | **95.4 (4.6)** | **96.7 (5.7)** | 93.9 (4.7) | 95.2 (5.2) | 97.5 (3.4) | 96.8 (6.3) | 95.3 (4.8) | 90.7 (8.9) |
|  | RF | 97.4 (5.2) | **94.0 (5.7)** | **95.8 (4.8)** | **97.4 (5.7)** | **94.3 (5.4)** | **95.6 (5.3)** | 98.2 (2.2) | 98.5 (2.0) | **95.7 (4.9)** | **91.4 (9.2)** |
|  | GB | 94.7 (7.9) | 91.5 (5.5) | 93.3 (4.7) | 94.9 (6.7) | 91.8 (5.7) | 93.0 (4.9) | **98.6 (1.9)** | **98.9 (0.0)** | 93.0 (5.2) | 86.4 (9.2) |
