# Supplementary material for: Predicting Abnormal Laboratory Blood Test Results in the Intensive Care Unit Using Novel Features Based on Information Theory and Historical Conditional Probability: Observational Study
Source: JMIR Med Inform. 2022 Jun 3;10(6):e35250. doi: 10.2196/35250 (PMC9206206; doi:10.2196/35250)
Supplement: Multimedia Appendix 3 [file medinform_v10i6e35250_app3.docx]

Multimedia Appendix 3. Percentual change for the ten-fold mean metric value between Approach 1 and Approach 2. An ^*^ means a statistically significant difference (two-sided Wilcoxon rank-sum hypothesis tests adjusted with Benjamini-Hochberg using a false positive rate set at 0.05). The difference was conducted for each classifier (FM: fuzzy model; LR: logistic regression; RF: random forest; GB: gradient boosting; BI: Bayesian inference) and for each metric (Sp.: specificity; Se.: sensitivity; Ac.: accuracy; Pr.: precision; NPV: negative predictive value; F1-score; AUC; PR AUC; Gmean; IBA).

| Lab. test | Model | Sp. | Se. | | Ac. | Pr. | NPV | F1 | AUC | PRAUC | G  mean | IBA |
| --- | --- | --- | --- | --- | --- | --- | --- | --- | --- | --- | --- | --- |
| PH Art. | FM | 5.9^*^ | | 8.0^*^ | 7.1^*^ | 5.1^*^ | 9.2^*^ | 6.7^*^ | 3.5^*^ | 3.0^*^ | 6.9^*^ | 14.5^*^ |
|  | LR | 11.6^*^ | | 2.6^*^ | 6.4^*^ | 7.9^*^ | 5.1^*^ | 5.1^*^ | 5.7^*^ | 4.3^*^ | 6.9^*^ | 13.6^*^ |
|  | RF | 7.8^*^ | | 5.4^*^ | 6.5^*^ | 6.0^*^ | 7.2^*^ | 5.7^*^ | 5.5^*^ | 4.4^*^ | 6.6^*^ | 13.5^*^ |
|  | GB | 5.1^*^ | | 7.8^*^ | 6.5^*^ | 4.6^*^ | 8.6^*^ | 6.2^*^ | 5.2^*^ | 4.0^*^ | 6.5^*^ | 13.3^*^ |
| PO2Art. | FM | -2.2 | | 2.2 | 0.3 | -0.3 | 0.7 | 0.7 | 0.4 | 1.3^*^ | -0.3 | -0.2 |
|  | LR | 5.8^*^ | | 5.0^*^ | 5.4^*^ | 5.1^*^ | 5.7^*^ | 5.0^*^ | 13.1^*^ | 7.9^*^ | 5.5^*^ | 11.0^*^ |
|  | RF | 2.3 | | 0.9 | 1.5^*^ | 1.6^*^ | 1.7 | 1.2 | 2.4^*^ | 1.8^*^ | 1.5^*^ | 3.0^*^ |
|  | GB | 1.6^*^ | | 1.7 | 1.7^*^ | 1.6^*^ | 1.8^*^ | 1.7^*^ | 2.1^*^ | 1.5^*^ | 1.7^*^ | 3.5^*^ |
| PCO2Art. | FM | 3.7^*^ | | 11.4^*^ | 7.8^*^ | 5.0^*^ | 10.4^*^ | 8.4^*^ | 3.9^*^ | 2.6 | 7.6^*^ | 16.4^*^ |
|  | LR | 29.1^*^ | | 50.4^*^ | 39.8^*^ | 33.2^*^ | 46.1^*^ | 41.7^*^ | 41.0^*^ | 37.3^*^ | 39.4^*^ | 96.2^*^ |
|  | RF | 1.2^*^ | | 12.0^*^ | 6.6^*^ | 3.2^*^ | 9.8^*^ | 7.5^*^ | 6.1^*^ | 5.0^*^ | 6.3^*^ | 14.1^*^ |
|  | GB | -0.1 | | 12.8^*^ | 6.3^*^ | 2.3^*^ | 9.8^*^ | 7.7^*^ | 5.8^*^ | 4.8^*^ | 6.0^*^ | 13.7^*^ |
| K | FM | 10.7^*^ | | 23.3^*^ | 13.5^*^ | 28.3^*^ | 7.4^*^ | 26.6^*^ | 28.5^*^ | 45.2^*^ | 16.9^*^ | 37.7^*^ |
|  | LR | 29.8^*^ | | -4.0^*^ | 20.5^*^ | 31.3^*^ | 2.6^*^ | 17.4^*^ | 15.7^*^ | 17.1^*^ | 11.8^*^ | 22.3^*^ |
|  | RF | -2.6^*^ | | 9.7^*^ | -0.1 | 0.7 | 1.6 | 4.4 | 2.2 | 3 | 3.4^*^ | 7.5^*^ |
|  | GB | 1.3 | | 0.5 | 1 | 2 | 0.2 | 1.4 | 0.8 | 1.5 | 0.9 | 1.7 |
| HGB | FM | -1.2 | | 4.3^*^ | 3.9^*^ | -0.1 | 31.0^*^ | 2.1^*^ | 1.6 | 0.2 | 1.5 | 3.5 |
|  | LR | -11.7 | | 6.1^*^ | 5.1^*^ | -0.5 | 32.8^*^ | 2.9^*^ | 0.7 | 0.1 | -5.1 | -5.9 |
|  | RF | -4.9 | | 1.8^*^ | 1.4^*^ | -0.2 | 12.9 | 0.7^*^ | 0.7 | 0.1 | -1.6 | -2.7 |
|  | GB | -1.1 | | 2.3^*^ | 2.1^*^ | 0 | 15.5 | 1.2^*^ | 0.3 | 0 | 0.6 | 1.4 |
| Na | FM | 3.9^*^ | | 4.3^*^ | 4.1^*^ | 5.9^*^ | 2.7^*^ | 5.2^*^ | -2.3^*^ | -9.5 | 4.1^*^ | 8.4^*^ |
|  | LR | 55.4^*^ | | 52.1^*^ | 54.0^*^ | 80.2^*^ | 35.6^*^ | 66.2^*^ | 54.7^*^ | 49.3^*^ | 54.1^*^ | 135.6^*^ |
|  | RF | -0.1 | | 6.0^*^ | 2.1^*^ | 1 | 3.0^*^ | 3.4^*^ | 0.9 | 1.2 | 2.9^*^ | 6.2^*^ |
|  | GB | 2.4^*^ | | 2 | 2.2^*^ | 3.5 | 1.4 | 2.8^*^ | 1.1 | 1.9 | 2.3^*^ | 4.6^*^ |
| HCT | FM | -5.9 | | 11.0^*^ | 9.9^*^ | -0.3 | 63.7^*^ | 5.5^*^ | 2.2 | 0.3 | 2.2 | 5.9^*^ |
|  | LR | -8.8^*^ | | 6.7^*^ | 5.6^*^ | -0.5^*^ | 27.6^*^ | 3.2^*^ | 0.5 | 0 | -1.3 | -1.3 |
|  | RF | -4 | | 1.4^*^ | 1.1^*^ | -0.2 | 7.7 | 0.5^*^ | 0.6 | 0 | -1.3 | -2.2 |
|  | GB | -2.2 | | 2.2^*^ | 1.9^*^ | -0.1 | 11.3 | 1.1^*^ | 0.4 | 0.1 | -0.1 | 0.3 |
| WBC | FM | -5.5^*^ | | 10.1^*^ | 4.1^*^ | -1.7^*^ | 11.6^*^ | 4.2^*^ | -3.6^*^ | -3.1^*^ | 2.1^*^ | 5.3^*^ |
|  | LR | -5.7^*^ | | 23.7^*^ | 11.8^*^ | -0.6 | 25.9^*^ | 11.6^*^ | 11.8^*^ | 4.4^*^ | 8.0^*^ | 18.9^*^ |
|  | RF | -1.6 | | 4.7^*^ | 2.3^*^ | -0.2 | 5.8^*^ | 2.2^*^ | 1.8^*^ | 1.2^*^ | 1.5^*^ | 3.6^*^ |
|  | GB | -2.5^*^ | | 5.3^*^ | 2.3^*^ | -0.8 | 5.7^*^ | 2.4^*^ | 1.8^*^ | 1.2^*^ | 1.3 | 3.3 |
| CO2 | FM | -2.0^*^ | | 9.1^*^ | 2.7^*^ | -0.3 | 5.3^*^ | 4.3^*^ | -1.1^*^ | -2.5 | 3.4^*^ | 7.9^*^ |
|  | LR | 11.5^*^ | | 16.7^*^ | 13.8^*^ | 15.5^*^ | 12.6^*^ | 16.1^*^ | 18.9^*^ | 9.6^*^ | 14.3^*^ | 30.8^*^ |
|  | RF | -1 | | 4.6^*^ | 1.6^*^ | 0 | 2.8^*^ | 2.3^*^ | 1.8^*^ | 2.7^*^ | 1.9^*^ | 4.3^*^ |
|  | GB | 0.1 | | 3.4^*^ | 1.6^*^ | 0.9 | 2.4^*^ | 2.2^*^ | 1.4^*^ | 1.7^*^ | 1.7^*^ | 3.8^*^ |
| Creatinine | FM | -9.6^*^ | | 28.9^*^ | 16.7^*^ | -1.6^*^ | 46.5^*^ | 13.8^*^ | 3.2 | 0.8 | 8.1^*^ | 20.2^*^ |
|  | LR | -10.2^*^ | | 48.9^*^ | 27.5^*^ | -1.6^*^ | 60.9^*^ | 24.2^*^ | 24.9^*^ | 6.5^*^ | 15.7^*^ | 38.9^*^ |
|  | RF | -3.7^*^ | | 1.2 | -0.2 | -1.3^*^ | 1.3 | 0 | -0.9 | -0.5 | -1.3 | -2.3 |
|  | GB | -6.5^*^ | | 5.4^*^ | 2.0^*^ | -2.0^*^ | 8.0^*^ | 1.8^*^ | -0.2 | 0 | -0.7 | -0.3 |
| Urea | FM | -3.1^*^ | | 17.1^*^ | 8.1^*^ | -1.1 | 18.3^*^ | 8.1^*^ | 0.6 | 0.1 | 6.5^*^ | 15.3^*^ |
|  | LR | -5.5^*^ | | 20.4^*^ | 8.5^*^ | -2.8^*^ | 20.7^*^ | 8.9^*^ | 11.2^*^ | 4.2^*^ | 6.6^*^ | 16.1^*^ |
|  | RF | -0.3 | | 3.9^*^ | 2.1^*^ | 0.1 | 4.8^*^ | 2.0^*^ | 0.4 | 0.3 | 1.8^*^ | 3.8^*^ |
|  | GB | -1.7 | | 4.8^*^ | 2.1^*^ | -0.7 | 5.8^*^ | 2.1^*^ | 0.3 | 0.3 | 1.5^*^ | 3.8^*^ |
| Glucose | FM | -0.1 | | 3.5 | 0.4 | 1.6 | 0.6 | 2.5 | 2.9 | 4.6 | 1.7 | 3.6 |
|  | LR | 0.1 | | -3.6 | -0.4 | -1.7 | -0.4 | -2.6 | -0.9 | -4.2 | -1.7 | -3.8 |
|  | RF | 1.6 | | -4.3 | 0.8 | 3.2 | -0.4 | 0 | 1.8 | 7.6 | -1.5 | -3.3 |
|  | GB | 0.5 | | -0.3 | 0.4 | 1.3 | 0 | 0.6 | 0.5 | 4.6 | 0 | 0 |
| ALT | FM | -1 | | 6.2^*^ | 2.9^*^ | -0.6 | 6.6^*^ | 2.8^*^ | -0.1 | -0.2 | 2.6^*^ | 5.8^*^ |
|  | LR | -2.3^*^ | | 5.0^*^ | 1.7 | -1.8^*^ | 5.3^*^ | 1.6^*^ | -1.0^*^ | -0.6^*^ | 1.3 | 3.3^*^ |
|  | RF | 0.3 | | 0.7 | 0.6 | 0.3 | 1 | 0.5 | 0 | 0.1 | 0.5 | 1.2 |
|  | GB | 0.3 | | 1 | 0.6 | 0.3 | 1.1 | 0.6 | 0 | 0 | 0.6 | 1.3 |
| Bilirubin | FM | -2.7^*^ | | 4.4^*^ | 0.9 | -2.3^*^ | 3.6^*^ | 1.1 | -1.1^*^ | -1.8^*^ | 0.9 | 2.3 |
|  | LR | -1.5 | | 4.2^*^ | 1.3^*^ | -1.1 | 3.6 | 1.7 | -2.5^*^ | -1.1 | 1.2 | 3.1 |
|  | RF | -0.9 | | 2.1 | 0.7 | -0.7 | 1.8 | 0.8 | -0.1 | 0 | 0.7 | 1.5 |
|  | GB | -0.6 | | 2.2 | 0.9 | -0.4 | 1.9 | 1 | 0.3 | 0.4 | 0.9 | 2 |
| ALP | FM | -1.4 | | 26.3^*^ | 5.6^*^ | 0.4 | 7.7^*^ | 13.9^*^ | 4.4 | 4.5 | 12.0^*^ | 27.5^*^ |
|  | LR | 0.1 | | 28.5^*^ | 7.1^*^ | 5.3 | 8.8^*^ | 16.9^*^ | 22.1^*^ | 10.7^*^ | 13.4^*^ | 30.9^*^ |
|  | RF | 1.2 | | 3.2 | 1.7 | 2.9 | 1.2 | 3.1 | 0.8 | 1.7 | 2.2 | 4.5 |
|  | GB | 1.2 | | 1.4 | 1.2 | 2.7 | 0.5 | 2.1 | 0.5 | 1.3 | 1.4 | 2.7 |
| Alb.  Blood | FM | -2.5 | | 4.9^*^ | 3.2^*^ | -0.5 | 10.4 | 2.3^*^ | 0.2 | 0 | 1.2 | 3 |
|  | LR | -4.9 | | 4.8^*^ | 2.5 | -1.4 | 8.8 | 1.9 | -1 | -0.3 | -0.1 | 0.7 |
|  | RF | -0.1 | | 1.9 | 1.5 | 0 | 5 | 1 | 0 | 0 | 0.9 | 1.9 |
|  | GB | -1.7 | | 2.3 | 1.3 | -0.5 | 4.9 | 0.9 | 0.7 | 0.3 | 0.4 | 1.1 |
| AST | FM | -3.5 | | 6.6^*^ | 3.9^*^ | -1.1 | 14.8^*^ | 2.8^*^ | -0.9 | -0.3 | 1.4 | 3.8 |
|  | LR | -7.7^*^ | | 10.5^*^ | 5.3^*^ | -2.6^*^ | 21.6^*^ | 4.0^*^ | -0.3 | -0.1 | 1.1 | 3.7 |
|  | RF | 0 | | 1.2 | 0.9 | 0 | 2.9 | 0.5 | 0.7 | 0.3 | 0.5 | 1.4 |
|  | GB | 0 | | 1.4 | 1 | 0 | 3.3 | 0.6 | 0.2 | 0.1 | 0.6 | 1.4 |
| GGT | FM | 0.4 | | -0.1 | 0.2 | 0.1 | -0.1 | 0 | -1.8 | -1.7 | 0.2 | 0.4 |
|  | LR | 0.1 | | 4.9 | 2.6 | 0.1 | 4.1 | 2.8 | -1.3 | -1.9 | 2.7 | 5.7 |
|  | RF | 3.7 | | 1.2 | 2.4 | 3.1 | 0.6 | 2.4 | -0.1 | 0.1 | 2.7 | 4.8 |
|  | GB | 1 | | -4.4 | -1.6 | 0.4 | -4.1 | -1.9 | 0.5 | 1.1 | -1.6 | -3.7 |
