# Supplementary material for: Predicting Abnormal Laboratory Blood Test Results in the Intensive Care Unit Using Novel Features Based on Information Theory and Historical Conditional Probability: Observational Study
Source: JMIR Med Inform. 2022 Jun 3;10(6):e35250. doi: 10.2196/35250 (PMC9206206; doi:10.2196/35250)
Supplement: Multimedia Appendix 4 [file medinform_v10i6e35250_app4.docx]

Multimedia Appendix 4. Predictive rules for Fuzzy model. (BP: blood pressure; RR: respiration rate; HR: heart rate; ‘_entropy’ means that the feature is the conditional-based version)

| **Laboratory test** | **Predictive rule** |
| --- | --- |
| PH Art. | If *pre-test probability* is **< 46.3%** and *urineOutput_entropy* is **< 1.0 bits**, then:  current test is **abnormal** |
| PO2Art. | If *SpO* is **> 95.2%** and *pre-test probability* is **< 40.9%**, then:  current test is **abnormal** |
| PCO2Art. | If *HR* is **> 92.1 BPM** and *pre-test probability* is **< 47.9%**, then:  current test is **abnormal** |
| K | If *first value of the day* is **< 4.0 mmol/L** and *pre-test probability* is **< 72.9%**, then:  current test is **abnormal** |
| HGB | If *first value of the day* is **< 120.5 g/L** and *pre-test probability* is **< 36.8%** and  *HR_entropy* is **< 0.3 bits** and *BP_entropy* is **< 0.3 bits**, then:  current test is **abnormal** |
| Na | If *pre-test probability* is **< 67.2%**, then:  current test is **abnormal** |
| HCT | If *first value of the day* is **< 0.3** and *pre-test probability* is **< 19.5 %**, then:  current test is **abnormal** |
| WBC | If *pre-test probability* is **< 46.8%** and *urineOutput_entropy* is < **0.9 bits**, then:  current test is **abnormal** |
| CO2 | If *urineOutput* is < **141.3 mmol/L** and *pre-test probability* is < **60.5%** and  *HR_entropy* is > **1.0 bits**, then:  current test is **abnormal** |
| Creatinine | If *pre-test probability* is **< 44.2%**, then:  current test is **abnormal** |
| Urea | If *pre-test probability* is < **56.2%**, then:  current test is **abnormal** |
| Glucose | If *first value of the day* is **> 9.0 mmol/L** and *pre-test probability* is **< 81.5%** and  *RR_entropy* is **> 0.6 bits** and *diagnosisLabel_entropy* is > **0.6 bits**, then:  current test is **abnormal** |
| ALT | If *pre-test probability* is **< 10.7%**, then:  current test is abnormal |
| Bilirubin | If *pre-test probability* is < **63.1%**, then:  current test is **abnormal** |
| ALP | If *pre-test probability* is **< 82.7%**, then:  current test is **abnormal** |
| Alb. Blood | If *first value of the day* is **< 28.9 g/L** and *pre-test probability* is **< 33.8%**, then:  current test is **abnormal** |
| AST | If *first value of the day* is **> 54.6 U/L** and *pre-test probability* is **< 12.2%**, then:  current test is **abnormal** |
| GGT | If *pre-test probability* is **< 77.2%**, then:  current test is **abnormal** |
